# Parametric Study on Seismic Performance of FRP-RC Exterior Beam–Column Joints

**DOI:** 10.3390/polym18202590

**Published:** 2026-10-21

**Authors:** Jiming Li, Bu Wang, Jing Huang, Xinyi Wang, Peng Zhang, Yabo Wang

**Affiliations:** 1 School of Civil Engineering, Tsinghua University, Beijing 100084, China; li-jm22@mails.tsinghua.edu.cn; 2 Shaanxi Academy of Building Research Co., Ltd., Xi’an 710082, China; 3 School of Civil Engineering and Smart Construction, Chang’an University, Xi’an 710064, China; huangj0214@chd.edu.cn (J.H.); 2018328008@chd.edu.cn (X.W.); zp1995@chd.edu.cn (P.Z.); wangyb@chd.edu.cn (Y.W.)

**Keywords:** fiber-reinforced polymer reinforcements, beam–column joints, seismic performances, nonlinear finite element analysis, hysteretic model

## Abstract

A comprehensive and quantitative understanding of the seismic performance of fiber-reinforced polymer (FRP) reinforced concrete (FRP-RC) beam–column joints is still lacking. The issue is further complicated by a wide variety of FRP reinforcements. In this study, the challenge was investigated numerically and theoretically. A refined three-dimensional nonlinear finite element modeling approach was developed and validated against experimental results. The complex stress state of FRP reinforcements and the bond-slip behavior between FRP bars and concrete were reproduced. A total of 20 full-scale exterior beam–column joints were designed to quantify the effects of a series of parameters, including FRP type, beam shear–span ratio, the diameter of beam longitudinal bars, the diameter of beam-end stirrups, and the diameter and spacing of joint-panel stirrups. Satisfactory seismic performance was confirmed by the hysteretic responses of the specimens. The significant differences among the roles of the parameters were revealed. Especially, compared with their GFRP-RC counterparts, the ultimate loads and the related beam-end displacements of CFRP-RC specimens increased by up to 92.3% and 22.2%, respectively. Taking FRP type into account, a hysteretic model that can accommodate degradations in stiffness and strength was developed for the seismic design of FRP-RC beam–column joints.

## 1. Introduction

Although conventional reinforced concrete (RC) structures have been extensively used in civil engineering, the corrosion of steel reinforcements leads to durability concerns [1,2], such as the cracking and spalling of concrete and the deterioration of load-carrying capacity [3,4,5]. Consequently, the considerable costs for strengthening and rehabilitation are inevitable [6]. Conventional solutions, such as increasing the thickness of concrete cover, applying surface barrier materials and adopting protective coatings for reinforcements, can only delay the corrosion process to various extents [7,8].

Fiber-reinforced polymer (FRP) is not only lightweight and high-strength, but also essentially immune to corrosion. Hence, in developing a new generation of concrete frame structures with a fundamental improvement in durability, FRP reinforcements are considered as the most promising alternative to conventional steel reinforcements [9,10,11]. With long-term immersion tests, Cao et al. [12] found that the tensile strength of FRP bars decreased slightly after 180 days, and carbon fiber-reinforced polymer (CFRP) bars exhibited higher residual strength than glass fiber-reinforced polymer (GFRP) bars. Hao et al. [13] conducted chloride-salt wet–dry cycling tests on the concrete beams strengthened with externally bonded FRP materials. The results showed that the flexural capacity of the RC beams strengthened with CFRP plates and sheets decreased by only approximately 4–6% under the most adverse wet–dry cycling environment. Bai et al. [14] performed static loading and fatigue tests on CFRP-strengthened beams that had been in service for six years under natural temperature-humidity conditions. It was found that the ultimate load-carrying capacities of the beams were essentially consistent with their unexposed counterparts. Lu et al. [15] carried out durability tests on the concrete beams strengthened with prestressed CFRP plates under chloride-containing wet –dry cycling conditions. The test results indicated that the influence of 90-day wet–dry cycling on the flexural capacity of the strengthened beams was limited, except for some loss in prestress.

Over the past three decades, the application of FRP reinforcements in concrete structures has been continuously explored, particularly in the concrete frames that are the most common structural system [16]. However, the transversely isotropic and linear elastic behaviors of FRP reinforcements differ significantly from the mechanical behaviors of conventional steel reinforcements [17,18]. When FRP bars fail under tension, the internal fiber filaments would rupture, accompanied by resin peeling and explosive splitting, resulting in a distinctly brittle failure mode. Therefore, the analytical models and design approaches developed for conventional RC members are no longer applicable to FRP-reinforced concrete (FRP-RC) members. Furthermore, FRP reinforcements typically consist of the matrix phase of resin and the reinforcing phase of fibers, and their mechanical properties depend primarily on those of fibers [19,20]. Various fibers with significant differences have been used to make FRP reinforcements, such as carbon, glass and basalt fibers. This means that fiber type has a fundamental influence on the mechanical behavior of FRP reinforcements.

Currently, the research on FRP-RC frames has mainly focused on the simple components under various loading conditions, such as the flexural behavior of FRP-RC beams [21,22,23], the shear behavior of FRP-RC beams [24,25,26], the axial compression behavior of FRP-RC columns [27,28,29,30] and the seismic performance of FRP-RC columns [31,32,33]. Beam–column joints are the critical regions for the transfer and redistribution of internal forces in frame structures. Under seismic loading, beam–column joints must possess sufficient shear capacity to avoid the brittle failure that would prevent the development of beam-end plastic hinges [34,35]. For conventional RC beam–column joints subjected to seismic action, with the yield of steel reinforcements, the truss mechanism is sufficient to explain the shear mechanism at joint panels [36,37], and the continuous degradations in stiffness, strength and energy dissipation capacity have been clearly confirmed [38,39]. On the other hand, a comprehensive and quantitative understanding of the mechanical behaviors of FRP-RC beam–column joints, especially for their seismic performance, is still lacking. The issue is further complicated by the wide variety of fibers used for FRP reinforcements, i.e., the type of FRP reinforcements.

A few scholars have conducted pilot studies on the seismic performance of FRP-RC beam–column joints, and only GFRP reinforcements have been involved. By the cyclic loading tests of five specimens, Mady et al. [40] found that GFRP-RC beam–column joints could reach an inter–story drift ratio of 4.0% with relatively small residual deformation. Ghomi et al. [41] conducted cyclic loading tests on two GFRP-RC beam–column joints and reported that the specimens could achieve inter-story drift ratios of up to 8% without brittle failure. Guo et al. [42] indicated that the energy dissipation capacity was relatively low in the case of GFRP reinforcements. Hasaballa et al. [43] pointed out that a relatively high shear stress level would cause the premature failure of GFRP-RC joint panels. For GFRP-RC joint panels, Safdar et al. [44] proposed a configuration involving diagonal FRP bars and additional anchorage components. It was designed to improve joint ductility, mitigate stiffness degradation and enhance energy dissipation capacity. In addition to these discrete stirrup arrangements, Lin et al. [45] proposed a novel GFRP-RC beam–column joint incorporating an internal FRP tube in the joint region. The FRP tube was designed to confine the concrete in the joint core, enhance the shear resistance of the joint, and improve its energy dissipation capacity.

In this study, the challenge was investigated numerically and theoretically. The novelty of this work is reflected in three areas. First, a total of 20 full-scale exterior FRP-RC beam–column joints were designed to comprehensively quantify the effects of a series of parameters, including FRP type, the shear–span ratio of the beam, the diameter of beam longitudinal bars, the diameter of the stirrups at the beam end, the diameter of the stirrups at the joint panel and the stirrup spacing at the joint panel. To perform the parametric study, a refined three-dimensional nonlinear finite element modeling approach was developed and validated against experimental results. Solid elements were used to reproduce the complex stress state of FRP reinforcements, while a bond-slip constitutive law was introduced to simulate the interaction between FRP bars and concrete. Finally, taking the FRP type into account, a hysteretic model that can accommodate degradations in stiffness and strength was developed based on the results of the parametric study. The proposed model provides a basis for the seismic design of FRP-RC beam–column joints as well as frame structures.

## 2. Materials and Methods

### 2.1. Specimen Design

To clarify the roles of a series of parameters on the seismic performance of FRP-RC beam–column joints, a total of 20 FRP-RC beam–column joint specimens were designed. The cross-sectional dimensions for the column and beam of the specimens were 750 mm × 350 mm (height × width) and 550 mm × 350 mm (height × width), respectively. The thickness of concrete cover was 30 mm. The column was 4050 mm long, while the beam length was determined according to the value of the shear–span ratio (as described in detail below).

**Figure 1:**
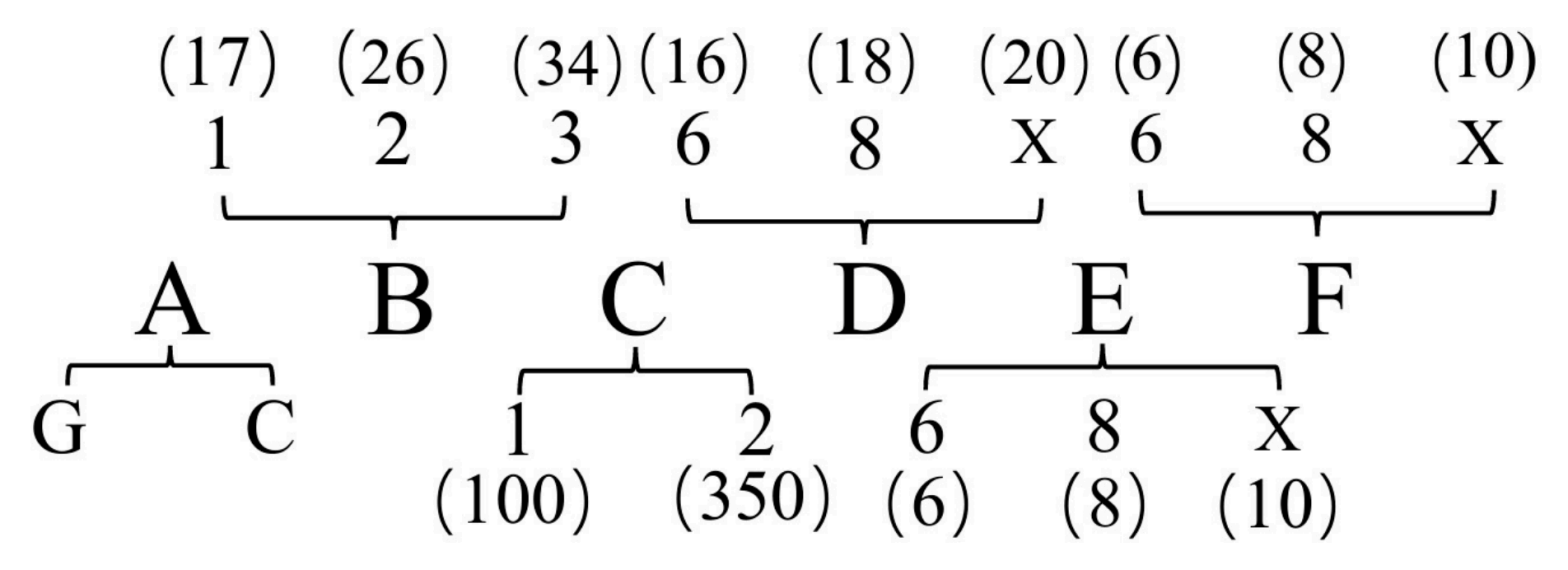
Specimen label.

The specimens were divided into two series, i.e., Series C and G, corresponding to CFRP and GFRP reinforcements, respectively. The former offers superior material properties and might represent the future direction in the field as its cost keeps dropping, whereas the latter is currently widely used. In each of the series, a set of parameters was considered, including the shear–span ratio of the beam, the diameter of beam longitudinal bars, the diameter of the stirrups at the beam end, the diameter of the stirrups at the joint panel and the stirrup spacing at the joint panel. Correspondingly, the specimens in each of the two series were further divided into 5 groups. The specimens were labeled as ABCDEF, as detailed in Figure 1 and Table 1. In the label, “A” was the name of series, i.e., “C” (CFRP) and “G” (GFRP); “B” denoted the shear–span ratio of the beam (*λ*); three practical “B” values, i.e., 1.7, 2.6, and 3.4, were selected; “C” referred to the stirrup spacing at the joint panel (*s*_vj_), and its values were 1 and 2 that corresponded to the spacing values of 100 mm and 350 mm, respectively; “D” denoted the diameter of beam longitudinal bars (*d*_b_); the “D” values were 6, 8 and “X” that corresponded to the diameters of 16 mm, 18 mm, and 20 mm, respectively; “E” represented the diameter of the stirrups at the beam end (*d*_vb_); three “E” values were 6, 8 and “X”, i.e., the diameters of 6 mm, 8 mm and 10 mm; “F” referred to the diameter of the stirrups at the joint panel (*d*_vj_); the “F” values were 6, 8 and “X” that corresponded to the diameters of 6 mm, 8 mm and 10 mm, respectively. For example, Specimen G12XXX represents a GFRP-RC beam–column joint, while the shear span ratio for the beam, the stirrup spacing at the joint panel, the diameter of beam longitudinal bars, the diameter of the stirrups at the beam end and the diameter of the stirrups at the joint panel were 1.7, 350 mm, 20 mm,10 mm and 10 mm, respectively.

### 2.2. FE Modeling

**Figure 2:**
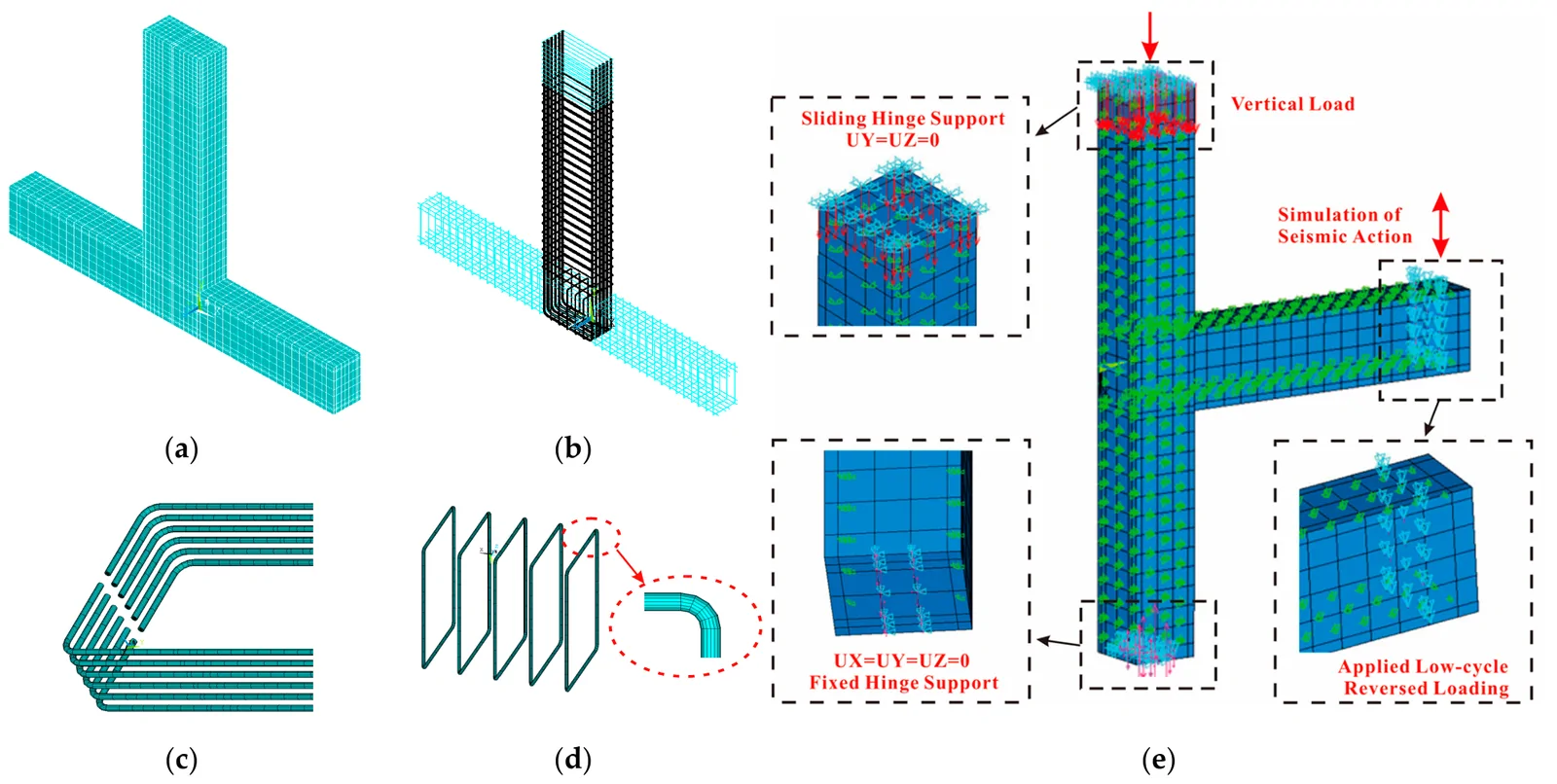
FE model: (**a**) concrete; (**b**) FRP reinforcements; (**c**) 3D modeling details of beam longitudinal FRP bars; (**d**) 3D modeling details of FRP stirrups; (**e**) boundary conditions.

The refined nonlinear full-scale 3D FE models of the specimens were built in ANSYS (version 14.5) [46], a large-scale, general-purpose FE analysis software package, as presented in Figure 2.

**Figure 3:**
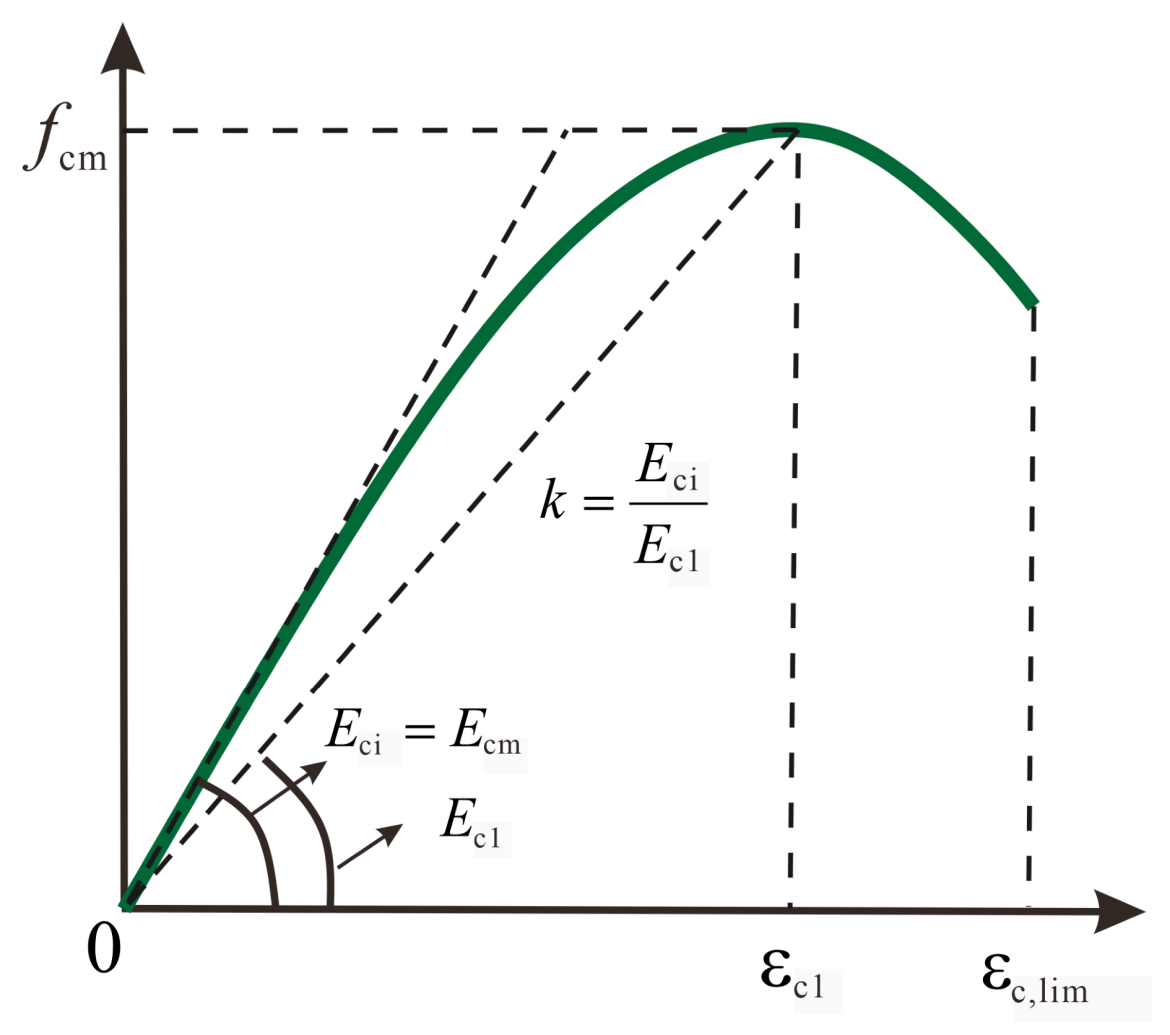
Stress–strain relationship for concrete.

Concrete was simulated by SOLID65 elements, as shown in Figure 2a. The solid elements can accurately capture the plastic deformation, cracking and failure of concrete [47,48,49,50]. Under cyclic loading, the stress–strain relationship of concrete was described by the constitutive model proposed by the International Federation for Structural Concrete (*fib*) [51], as presented in Equation (1) and Figure 3. The failure criterion for concrete followed the Willam–Warnke five-parameter failure criterion [52], which can effectively represent the tensile cracking and compressive crushing of concrete.

*σ*_c_*/f*_cm_ = −[(*kη* − *η*^2^)/(1 + (*k* − 2)*η*] (1)

where *η* = *ε_c_*/*ε_c_*_1_; *k* = *E_c_*/*E_c_*_1_; *f_cm_* is peak stress; *E_c_* is elastic modulus; *E_c_*_1_ is the secant modulus from the origin to the point corresponding to the peak stress; *ε_c_*_1_ is the strain corresponding to the peak stress; *ε_c_* is ultimate compressive strain.

As transversely anisotropic materials, FRP reinforcements exhibit high tensile strength and elastic modulus along the fiber direction. However, the properties at their cross sections are mainly governed by the resin matrix, which results in relatively low strength and the susceptibility to shear failure [53]. This means that the failure of FRP reinforcements depends not only on the axial stress of FRP reinforcements but also on the shear stress suffered by FRP reinforcements themselves. As a result, the two-node line elements (e.g., LINK8 elements), typically used for steel reinforcements to significantly simplify calculations [54,55,56], are no longer suitable for reproducing the complex mechanical behavior of FRP reinforcements. To avoid the consequences of excessive assumptions, 3D solid elements are the best choice. Therefore, SOLID64 elements were chosen for FRP reinforcements, as shown in Figure 2b–d. The anisotropic solid elements can describe the differences in material properties among different material directions.

**Figure 4:**
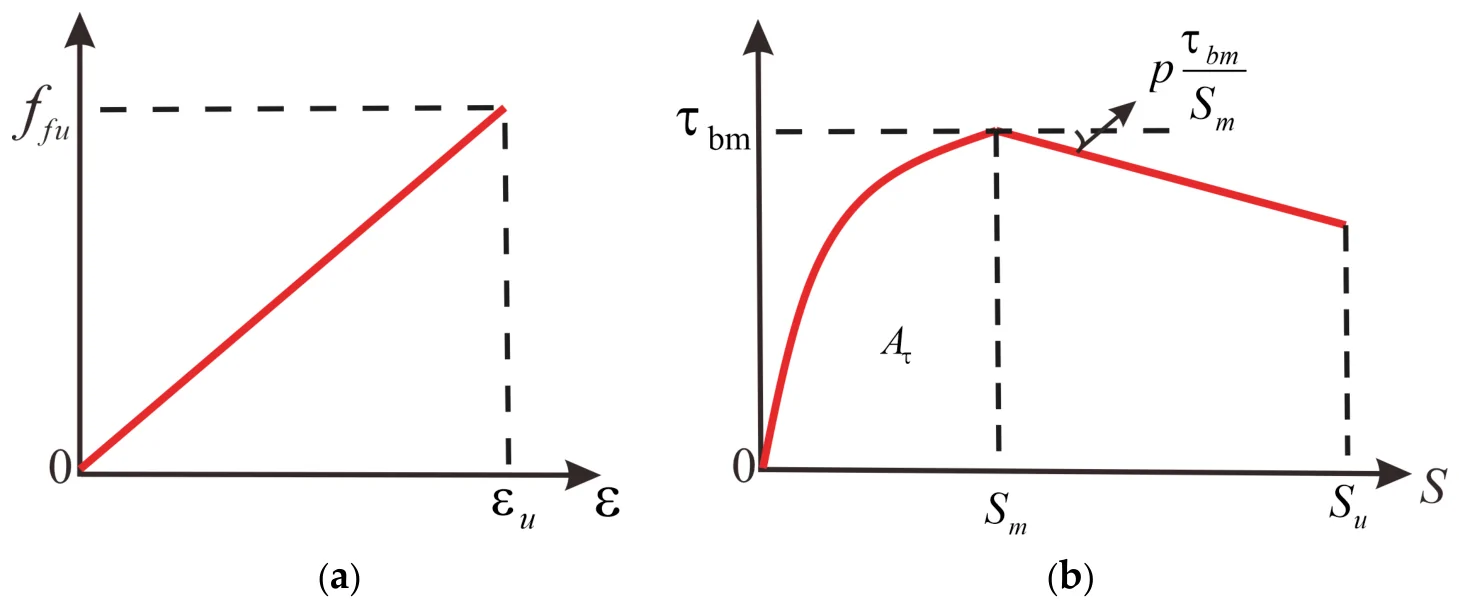
Material models for FRP: (**a**) constitutive relationship; (**b**) bond–slip relationship.

Compared with conventional steel reinforcements, a distinct yield plateau is lacking in the FRP case. Hence, FRP reinforcements are generally idealized as linear elastic materials, as shown in Figure 4a [57,58,59]. The three-dimensional linear-elastic behavior of the transversely isotropic FRP reinforcements can be defined by five independent engineering constants, namely longitudinal elastic modulus *E*_X_, transverse elastic modulus *E*_y_, Poisson’s ratios *ν*_xy_ and *ν*_yz_, and longitudinal shear modulus *G*_xy_ and *G*_yz_. In this study, the engineering constants were determined by the classic Chamis micromechanical model [60], using the mechanical properties and volume fractions of the fiber and resin constituents [61,62]. A fiber volume fraction of 60% was assumed. The calculated engineering constants for CFRP and GFRP reinforcements are listed in Table 2. To further accurately represent the interaction mechanism at the longitudinal FRP bar–concrete interface, the bond–slip constitutive model proposed by the International Federation for Structural Concrete (*fib*) [51] was adopted, as shown in Figure 4b.

In addition, SOLID64 and SOLID65 elements have compatible mesh sizes and identical nodal degrees of freedom (i.e., the three linear displacements in the x-, y- and z-directions). Furthermore, COMBIN39 nonlinear spring elements were introduced to simulate the non-uniform distribution of bond stress along the longitudinal FRP bars. The two ends of each of the elements were connected to an FRP node and a concrete node, respectively. The two nodes were located at the same position at the FRP–concrete interface.

As a result of the mesh-convergence study, the global mesh size for the FE models was 100 mm. To coordinate with the thickness of concrete cover and the locations of FRP bars and stirrups, the meshes for the FE models were further refined to various extents at the corresponding zones.

It should also be noted that, for specimens with relatively dense longitudinal bars and strips and in the case of round-section reinforcements, generating each individual reinforcement element would significantly increase the complexity of FE meshes and inevitably lead to mesh distortion. To address this issue, geometric overlap was adopted to improve the robustness of the simulation. In the previous studies [63,64,65], the impact of this simplification on the accuracy of FE simulation has been shown to be acceptable.

**Figure 5:**
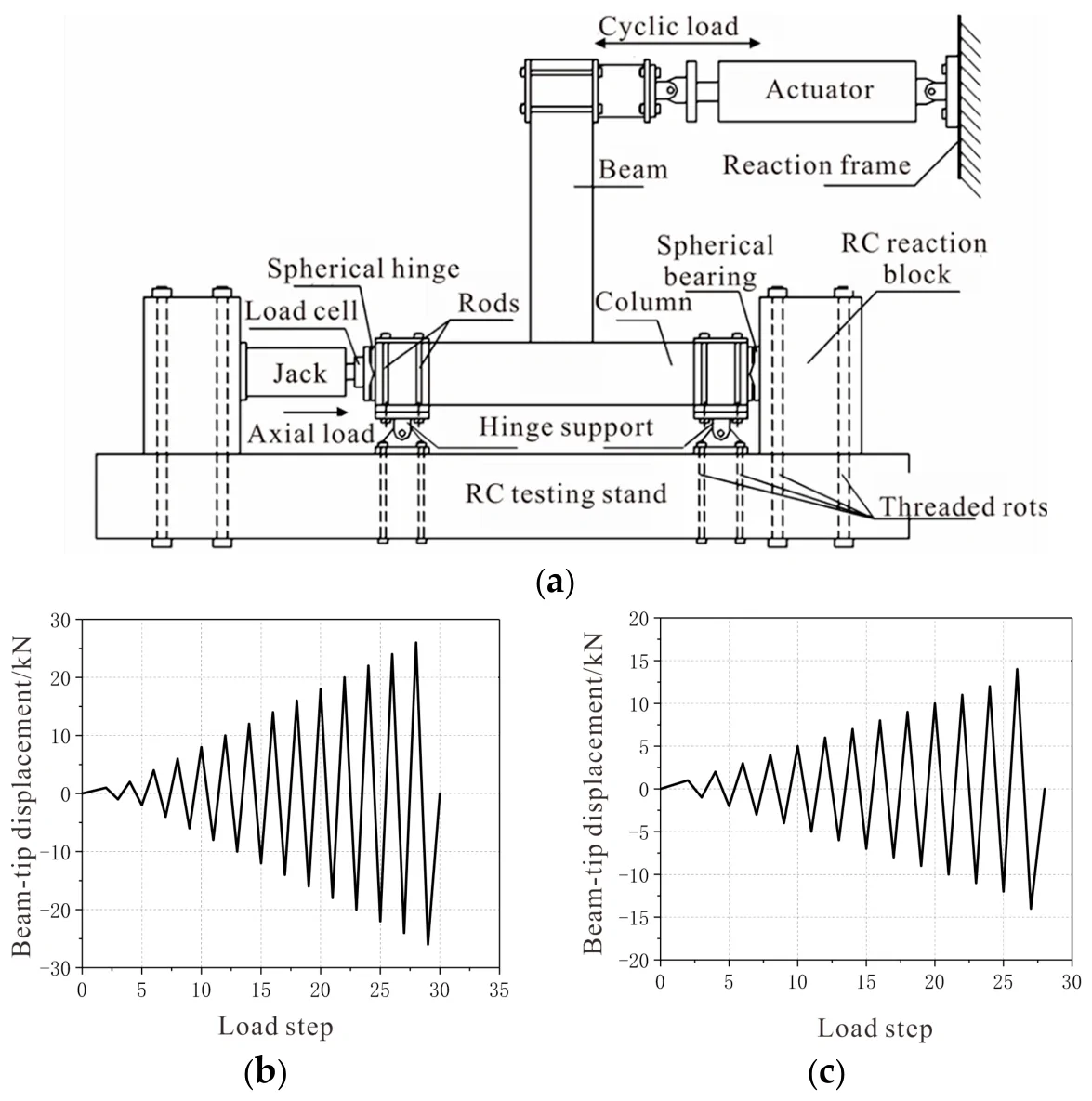
Test method: (**a**) test setup [24]; (**b**) displacement history I; (**c**) displacement history II.

To reproduce the loading pattern adopted in the tests of Ref. [24] (see Figure 5a), the specimens were constrained and loaded with the boundary conditions shown in Figure 2e. A fixed hinge was applied at the bottom surface of the column, i.e., the middle two rows of nodes were constrained in the X-, Y-, and Z-directions. An axial load was applied at the top surface of the column. Correspondingly, a sliding hinge was formed at the top surface of the column. Its difference from the fixed hinge was that only lateral displacements of the related nodes were restrained. Moreover, the nodes at the cross section located 50 mm from the beam tip were coupled, where reversed cyclic load was applied.

### 2.3. Loading Protocol

The cyclic loading process consisted of two stages. In the first stage, an axial load of 134.8 kN was applied at the column to represent dead and service Loads. In the second stage, a cyclic load was applied at the beam tip to simulate seismic action. The axial load remained unchanged during the cyclic loading. The cyclic loading was performed following a displacement-controlled regime. Furthermore, according to the different beam-end shear–span ratios, two loading protocols were designed. For the specimens with the shear–span ratios of 2.6 and 3.4, the increment for load steps was 2 mm, as shown in Figure 5b. In the case of the relatively small shear–span ratio of 1.7, shear-dominated behavior was expected. Therefore, a smaller increment of 1 mm was applied to the load steps to improve the stability and convergence of the nonlinear FE analysis, as shown in Figure 5c. Failure was identified when the average of the peak loads reached in the forward and reverse directions in a load step decreased to 85% of the ultimate one.

### 2.4. Model Validation

The experimental results of two T-shaped exterior beam–column joints, i.e., specimens JFRP1 [66] and GG–3 [40], were selected to validate the proposed finite element (FE) modeling approach. Specimens JFRP1 and GG–3 were CFRP-RC and GFRP-RC specimens, respectively.

**Figure 6:**
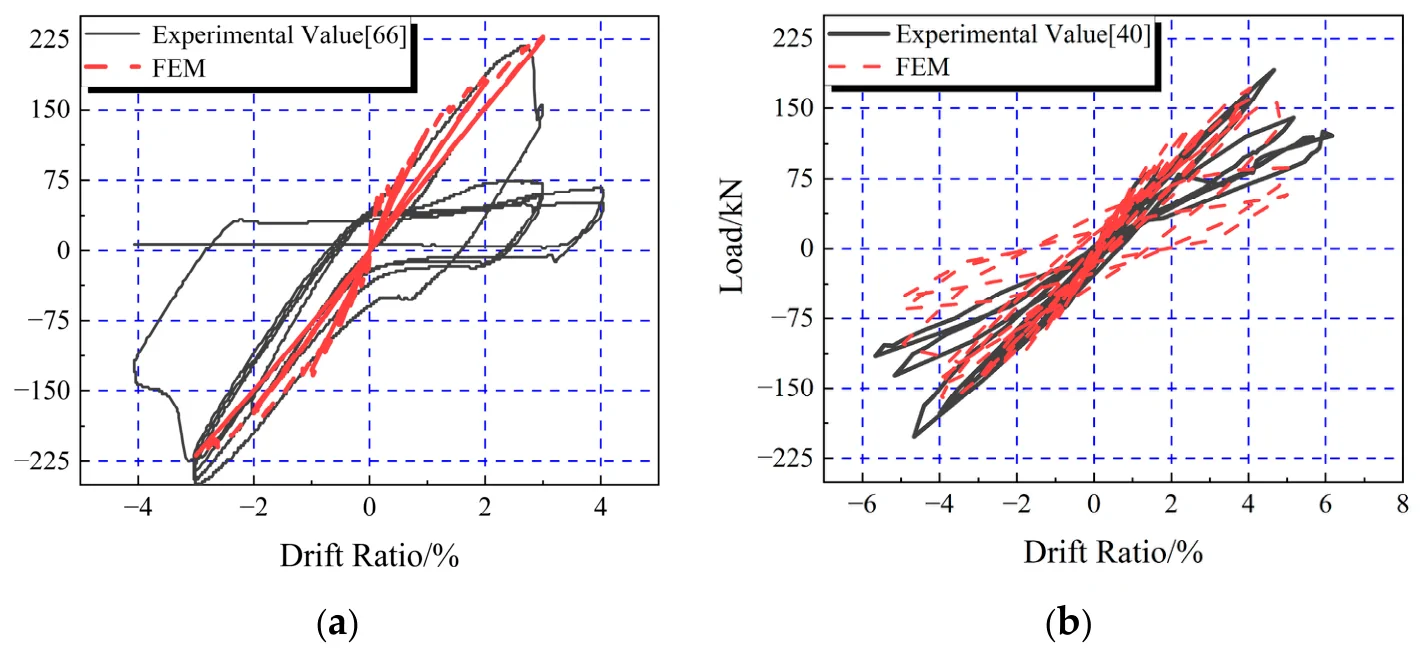
Comparison of numerical and experimental hysteretic curves: (**a**) Specimen JFRP1 [66]; (**b**) Specimen GG–3 [40].

The comparison between the numerical and experimental hysteresis curves of Specimen JFRP1 is presented in Figure 6a. It can be observed that the numerical curve agrees well with its experimental counterpart, with nearly identical ultimate loads. In Specimen GG–3 case, the numerical result is also consistent with the experimental one, as shown in Figure 6b. The consistency between the numerical and experimental results is further confirmed by the comparison of the key indicators of the hysteretic responses listed in Table 3, namely average maximum load and average displacement at maximum load. The above observations imply that an effective cooperation between the solid-modeled FRP and concrete was achieved in the proposed FE modeling approach.

**Figure 7:**
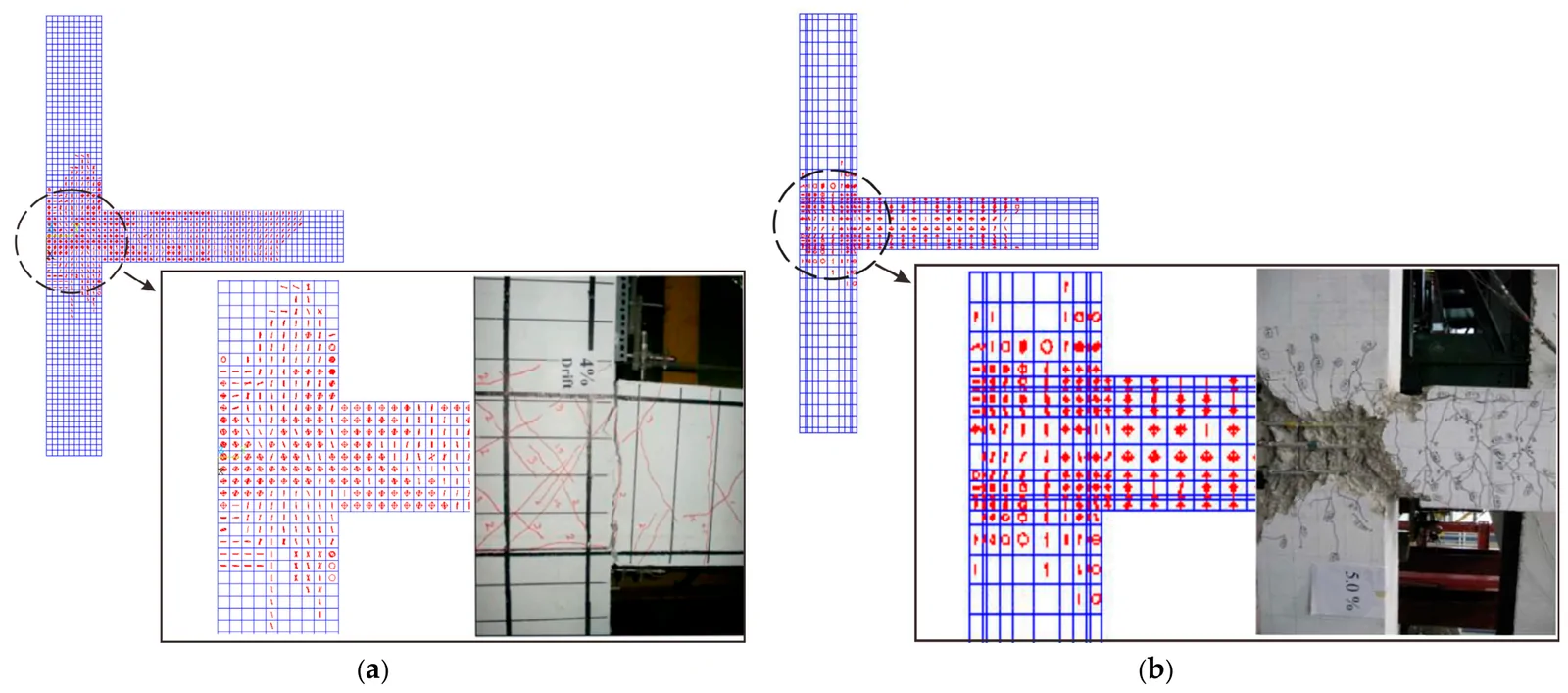
Comparison of numerical and experimental failure modes: (**a**) Specimen JFRP1 [66]; (**b**) Specimen GG–3 [40].

For Specimens JFRP1 and GG–3, the simulated failure patterns and their experimental counterparts are compared in Figure 7a,b, respectively. The cracking and crushing of concrete are indicated by the short lines and diamond symbols marked at element centroids. For each of the specimens, both the numerical and experimental results clearly indicate the shear failure of the joint panel. The diagonal cracking and crushing phenomena at the joint panels were accurately reproduced by simulation. 

The above findings confirmed the effectiveness of the proposed FE modeling approach in predicting the seismic performance of FRP-RC beam–column joints. In particular, the 3D modeling technique for FRP reinforcements is robust.

## 3. Results and Discussion

### 3.1. Failure Modes

**Figure 8:**
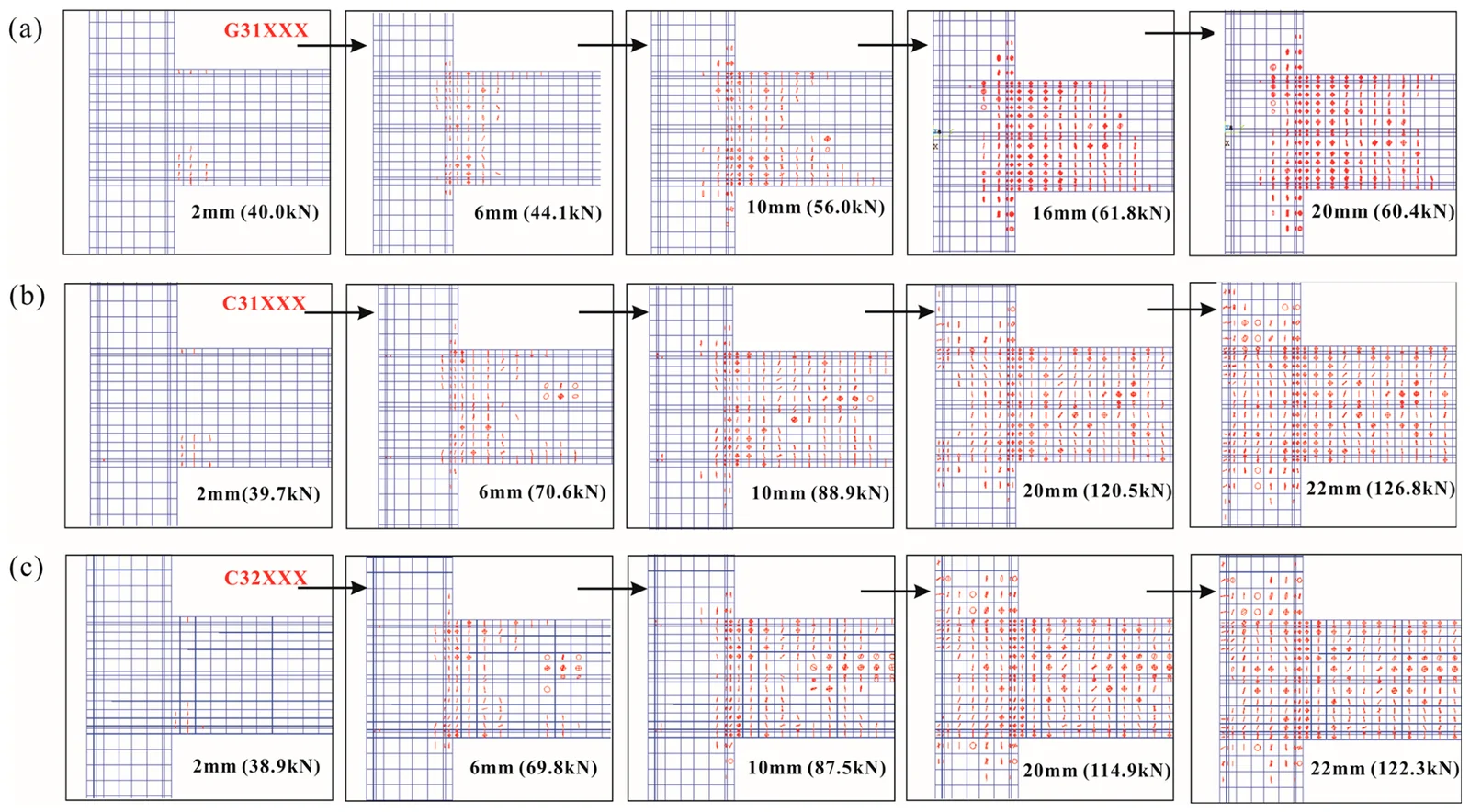
Concrete damage patterns: (**a**) Specimen G31XXX; (**b**) Specimen C31XXX; (**c**) Specimen C32XXX.

The failure modes of the specimens, represented by the concrete damage patterns of Specimens G31XXX, C31XXX and C32XXX, are presented in Figure 8. The values in parentheses represent the average peak loads of the current load steps.

As shown in Figure 8a, in the case of GFRP reinforcements, the extent of concrete damage in Specimen G31XXX increased with the beam-tip control displacement. At the beam-tip control displacement of 2 mm, vertical cracks (i.e., the short vertical lines) appeared at the beam end, which implies that the specimen had entered the nonlinear state dominated by flexural damage. When the beam-tip control displacement was increased to 6 mm, diagonal shear cracks (i.e., the inclined short lines) appeared at the middle of the web of the beam end. That is, the flexural-shear cracks at the beam end developed significantly. Meanwhile, some short, nearly vertical cracks appeared at the upper edge of the joint panel on the beam side. This is obviously due to the excessive principal tensile stress induced by anchoring the beam FRP longitudinal bars, which are subjected to tensile force. A similar phenomenon is commonly observed in the case of conventional RC beam–column joints after yielding of the beam steel longitudinal bars, and is referred to as yield penetration. It often occurs before the formation of diagonal shear cracks and has a pronounced influence on the integrity of the joint panel. Therefore, it may be regarded as a triggering factor for the shear failure of the joint panel. The above observation indicates that the effect similar to yield penetration did not disappear as conventional steel bars were replaced by FRP ones. Considering the linear elastic behavior of FRP bars, the pseudo-yield penetration effect caused by beam longitudinal FRP bars might lead to a more severe consequence. As the beam-tip control displacement was increased to 10 mm, the flexural-shear cracks at the beam end developed significantly. Especially, the crushing of concrete occurred within a region extending outward from the beam–column interface with a length approximately equal to the beam depth. At the same time, the cracking region at the joint panel expanded markedly toward the middle, accompanied by the phenomenon of concrete crushing at the edge on the beam side. As the beam-tip control displacement was further increased to 20 mm, the specimen reached its ultimate load. Concrete at the beam end was severely crushed, whereas the damage range of the joint panel extended very slowly. This indicates that the specimen experienced the beam-end flexural failure governed by the concrete crushing at the compression zone.

Compared with its GFRP-RC counterpart (i.e., Specimen G31XXX), the CFRP-RC specimen C31XXX suffered beam-end cracking, beam-end crushing, and joint-panel cracking for the first time at the same beam-tip control displacements. The ratios of the average peak load to the ultimate load were also approximately the same at the load steps where the phenomena appeared. However, at the occurrence of the above phenomena, the beam-tip loads for Specimen C31XXX were 2%, 14.3%, and 37.8% higher than those for Specimen G31XXX, respectively. For this reason, as the beam-tip control displacement was increased from 6 mm to 10 mm, the flexural damage range at the beam end of Specimen C31XXX was significantly larger than that of Specimen G31XXX. On the other hand, the damage at the joint panel of the former was less severe than that of the latter. This might be attributed to the more effective confinement provided by the CFRP stirrups that possessed a much higher elastic modulus. At the beam-tip control displacement of 20 mm, i.e., the load step corresponding to the ultimate load of Specimen G31XXX, Specimen C31XXX behaved differently from Specimen G31XXX. While Specimen G31XXX failed due to the concrete crushing at the beam end, extensive cracking occurred at the joint panel of Specimen C31XXX. This implies that the shear force of the joint panel was substantially increased by the significant increase in the tensile force of the beam longitudinal CFRP bars. Meanwhile, the compression zone of the beam end was effectively confined by CFRP stirrups; the crushing of concrete was delayed. Especially, Specimen C31XXX reached only 88% of its ultimate load at this load step. As the beam-tip displacement was further increased to 22 mm, the concrete crushing at the beam end became very severe. The failure of the CFRP-RC specimen was characterized by a combined mode involving the concrete crushing at the beam end and the diagonal cracking at the joint panel. The difference in failure modes between Specimens C31XXX and G31XXX indicates a potential safety concern arising from the linear elastic behavior of FRP reinforcements. That is, although the high elastic modulus of CFRP bars significantly improved the flexural capacity of the beam end, the unexpected excessive shear demand might be imposed on the joint panel. Therefore, besides force equilibrium, deformation compatibility should be considered in establishing the strength hierarchy for the different components of an FRP-RC beam–column joint.

The comparison between Figure 8b,c shows that the shear damage at the joint panels of CFRP-RC beam–column joints became more severe as the spacing of the joint stirrups was increased. At the beam-tip control displacements of no more than 10 mm, increasing the stirrup spacing from 100 mm to 350 mm had no significant effect on the range of concrete cracking at the joint panel. At this stage, the cracks at the joint panel were mainly concentrated on the side close to the beam end and corresponded to the locations of beam longitudinal bars. It is primarily the result of the aforementioned pseudo-yield penetration effect caused by beam longitudinal bars, while the shear resistance of the joint panel is dominated by the diagonal concrete compression strut. At the beam-tip control displacement of 20 mm, significant cracking occurred at the joint panels of both Specimens C31XXX and C32XXX, which enabled FRP stirrups to play an important role in the shear mechanism of the joint panels. As a result, in comparison with its 100 mm-spacing counterpart (i.e., Specimen C31XXX), the joint panel of Specimen C32XXX not only exhibited a larger cracking range but also suffered relatively severe concrete crushing. As the beam-tip control displacement was further increased to 22 mm, at the joint panel of Specimen C32XXX, extensive concrete crushing occurred along the diagonal direction (i.e., along the concrete compression strut). This indicates that the joint panel was approaching its shear limit. On the other hand, no significant concrete crushing is observed at the joint panel of Specimen C31XXX. This indicates that the stirrup spacing of 100 mm is acceptable. Meanwhile, at the beam-tip control displacements of 20 mm and 22 mm, the average peak loads of Specimen C32XXX were always slightly lower than their counterparts for Specimen C31XXX. This is obviously due to the fact that the shear deformation of the joint panel accounted for a larger proportion of the overall deformation in the case of Specimen C32XXX. As can be seen from the above analysis, the effect of the spacing of FRP stirrups on the shear performance of joint panels is similar to that of conventional steel stirrups. However, considering the unexpected excessive joint shear force caused by the linear elastic beam longitudinal FRP bars, the design of FRP joint stirrups should be more conservative, particularly in the case of high-modulus CFRP ones.

### 3.2. Hysteretic Responses

**Figure 9:**
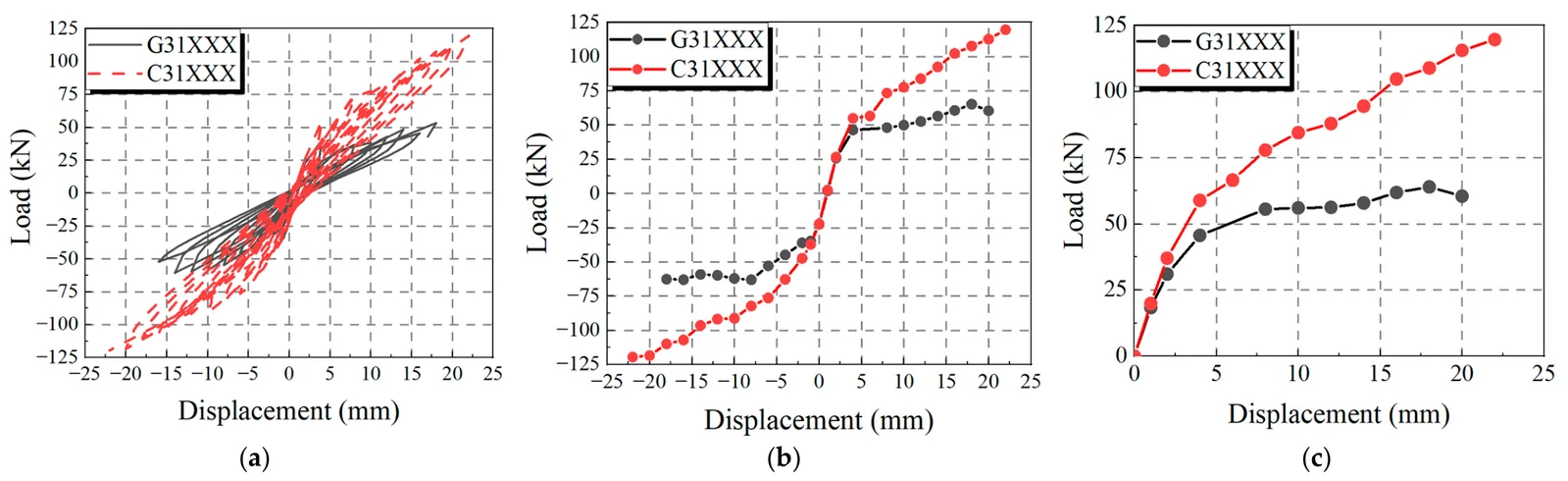
Comparison of hysteresis responses of Specimens G31XXX and C31XXX: (**a**) hysteretic curves; (**b**) envelope curves; (**c**) average envelope curves.

**Figure 10:**
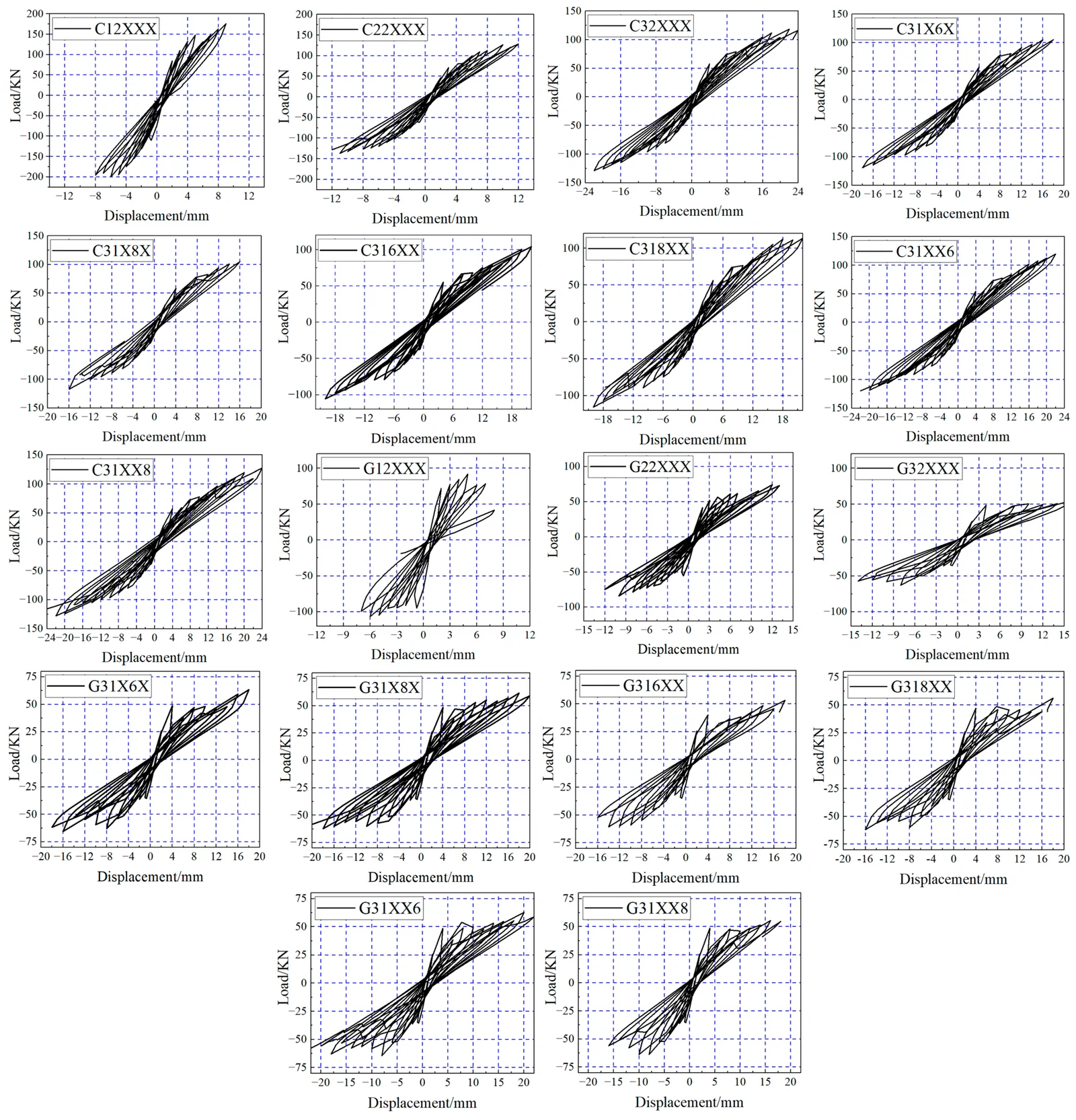
Hysteresis curves.

The hysteretic responses of FRP-RC beam–column joints are discussed with the results of the aforementioned typical specimens, i.e., Specimens G31XXX and C31XXX. The hysteretic curves and envelope curves of the two specimens are compared in Figure 9a,b, respectively. The averaged envelope curves are illustrated in Figure 9c. For the averaged envelope curves, the beam-tip load of each load step was the average of the peak loads reached in the forward and reverse directions. The hysteretic curves of other specimens are shown in Figure 10.

As shown in Figure 9a, before the beam-tip control displacement reached 5 mm, the loading and unloading branches of the hysteretic loops almost overlapped. With the development of cracks, however, the two branches gradually separated, which exhibits a distinct characteristic of hysteretic response. In addition, a pronounced pinching phenomenon is exhibited by the hysteretic curves, which corresponds to the damage of concrete crushing at the beam end and the shear cracking at the joint panel. It should also be noted that, under the same beam-tip control displacement, the hysteretic loop area for Specimen C31XXX is significantly larger than that of Specimen G31XXX. This is mainly because CFRP reinforcements have a much higher elastic modulus relative to their GFRP counterparts. Hence, not only lateral stiffness but also peak load was much greater in the case of CFRP-RC beam–column joints. It is concluded that, considering that the expected failure mode for FRP-RC beam ends is the concrete crushing with relatively low ductility, the energy dissipation of FRP-RC joints depends mainly on their load-carrying capacities. It was also noted that the shape of the hysteretic loops of Specimen C31XXX was fuller than that of Specimen G31XXX during the process of approaching ultimate load. This implies that relatively severe non-ductile damage occurred in Specimen C31XXX. As shown in Figure 8, besides the beam-end flexural damage similar to that experienced by Specimen G31XXX, Specimen C31XXX suffered extensive shear damage at its joint panel due to the significantly increased tensile force of its beam longitudinal CFRP bars.

As presented in Figure 9b,c, the envelope curves of the two specimens almost overlap with each other at the load step of 2 mm. As shown in Figure 8a,b, no cracking occurred during this stage. This indicates that the specimens remained in an elastic state. That is, the mechanical behavior of the specimens was governed by concrete. As the beam-tip control displacement was further increased, the envelope curves exhibited obvious nonlinear characteristics. In each of the plots, Curve C31XXX was significantly higher than Curve G31XXX, and the difference between the two curves increased with the beam-tip control displacement. Finally, the average ultimate load of Specimen C31XXX was approximately 92.3% higher than that of Specimen G31XXX, while the corresponding beam-end displacement increased by 22.2%. In particular, distinctly different mechanical characteristics are reflected by the nonlinear segments of the envelope curves. First, except for several minor fluctuations, the nonlinear segments of Curve C31XXX show a rapidly increasing trend while secant stiffness decreased gradually. As the beam-tip control displacement was increased from 4 mm to 22 mm, the average peak load increased by 103.3%. At failure, the residual secant stiffness was still 27.4% of the initial stiffness. This reflects the significant effect of high-modulus, linear-elastic CFRP reinforcements on improving the seismic performance of FRP-RC beam–column joints. By contrast, the nonlinear segments of Specimen G31XXX present a very slowly increasing trend. At the load steps from 5 mm to 20 mm, the average peak load increased by only 32.5%, which exhibits an evident pseudo-yielding characteristic. The cooperative mechanism between low-modulus GFRP longitudinal bars and concrete is demonstrated by this phenomenon. Although GFRP is also linear elastic, the full use of the strength of GFRP longitudinal bars depends on the excessive flexural deformation of the beam end. Consequently, a rapid degradation of the compression zone at the beam end was inevitable with concrete crushing. During the process of stress redistribution over the beam cross section, the increment in the contribution of GFRP longitudinal bars to flexural capacity is counteracted by the negative effect from the reduction in moment arm. It should be noted that, due to the expected beam-end failure mode of concrete crushing, the pseudo-yielding ductility of GFRP-RC bars is not necessarily accompanied by the rounded hysteretic loops that were typically observed in the case of conventional RC beams. Therefore, in the case of low-modulus FRP reinforcements, the seismic performance related to the pseudo-yielding flexural failure should not be evaluated with the ductility factor adopted for conventional RC structures.

### 3.3. Concrete Stress Distribution

**Figure 11:**
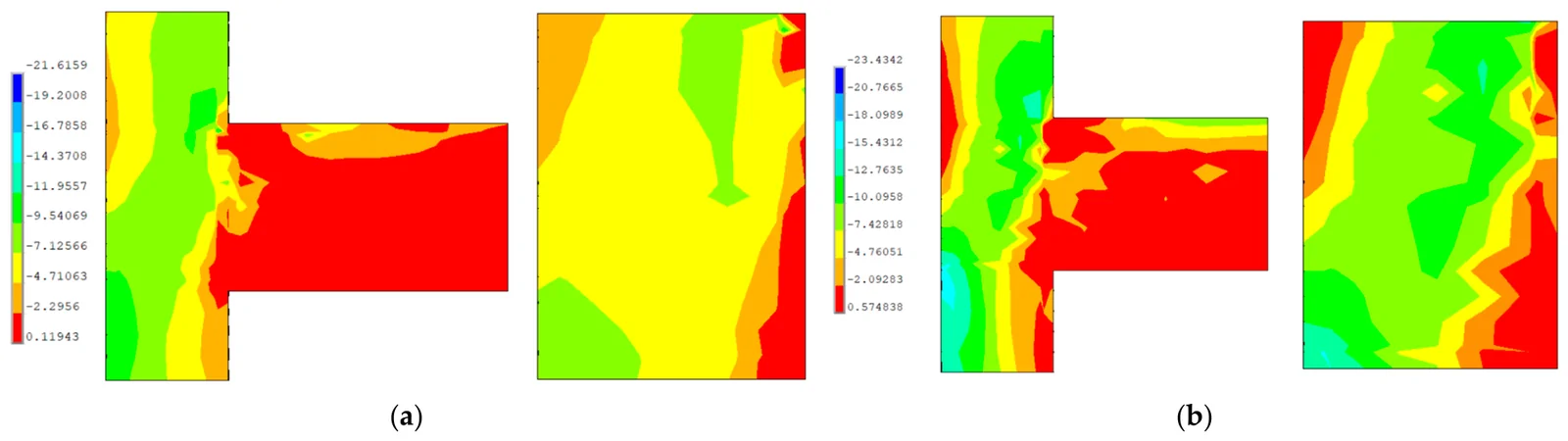
Concrete principal compressive stress distribution at ultimate load: (**a**) Specimen G31XXX; (**b**) Specimen C31XXX.

For the typical specimens in Series G and C (i.e., Specimens G31XXX and C31XXX), the concrete principal compressive stress distributions corresponding to their respective ultimate load are presented in Figure 11a,b.

As shown in Figure 11, the stress distributions at the beam and column ends conform to the typical bending-dominated pattern. Moreover, a distinct diagonal compression strut appears at the joint panel of each of the specimens. This indicates that the diagonal compression mechanism plays a key role in resisting the shear force at the joint panel. The reason is twofold. On the one hand, the conventional truss mechanism based on steel yielding cannot be formed due to the linear-elastic behavior of FRP stirrups. On the other hand, the damage in both specimens occurred primarily at the beam ends. As a result, the nonlinear response of the joint panels was relatively limited, which is evidenced by the fact that the maximum principal compressive stress values of both the joint panels were far below the compressive strength of concrete. Under the ultimate limit state for shear, a strut-tie mechanism is most likely to be formed at the FRP-RC joint panel, i.e., the compression struts of concrete and the tension ties consisting of FRP reinforcements. In addition, the stress values at the ends of the diagonal compression struts are significantly higher than those at the middle, which is obviously caused by the stress concentrations resulting from the changes in force and geometry at the beam–column interfaces.

Meanwhile, between Specimens G31XXX and C31XXX, a noticeable difference in the principal compressive stress distribution in the diagonal compression strut at the joint panel could be discerned. First, except for the stress concentration at the two ends, the principal compressive stress for the diagonal compression of the GFRP-RC specimen was approximately in the range of approximately 5 MPa to 11 MPa, whereas that of the CFRP-RC specimen was as high as 18 MPa. This is evidently due to the higher joint shear force in the CFRP-RC specimen case, i.e., the ultimate load of Specimen C31XXX is 1.98 times that of Specimen G31XXX (see Table 1). On the other hand, it demonstrates that the joint panel of the GFRP-RC specimen was essentially in an elastic state (see Figure 8a). Furthermore, it should be noted that the diagonal compression strut of Specimen C31XXX is significantly narrower than that of Specimen G31XXX.

In addition, noticeable disturbances exist in the principal compressive stress contour map for the diagonal compression strut of Specimen C31XXX. The phenomenon reflects that the damage at the joint panel was relatively severe under substantially increased shear force.

### 3.4. Stress Distribution of FRP Reinforcements

**Figure 12:**
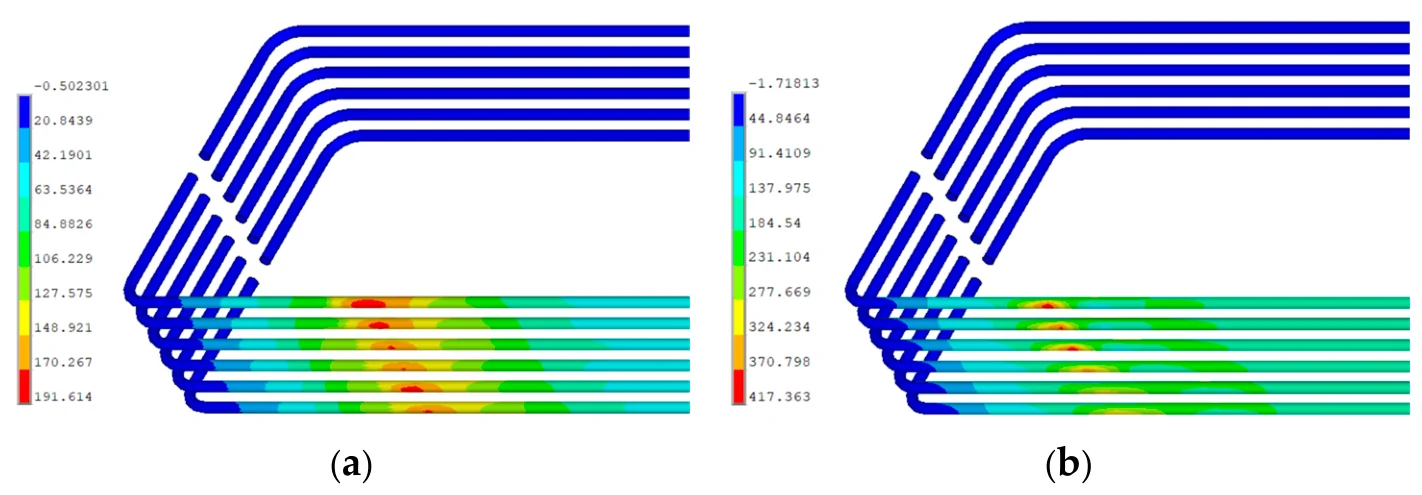
Principal tensile stress distribution of beam longitudinal FRP bars under ultimate load: (**a**) Specimen G31XXX; (**b**) Specimen C31XXX.

**Figure 13:**
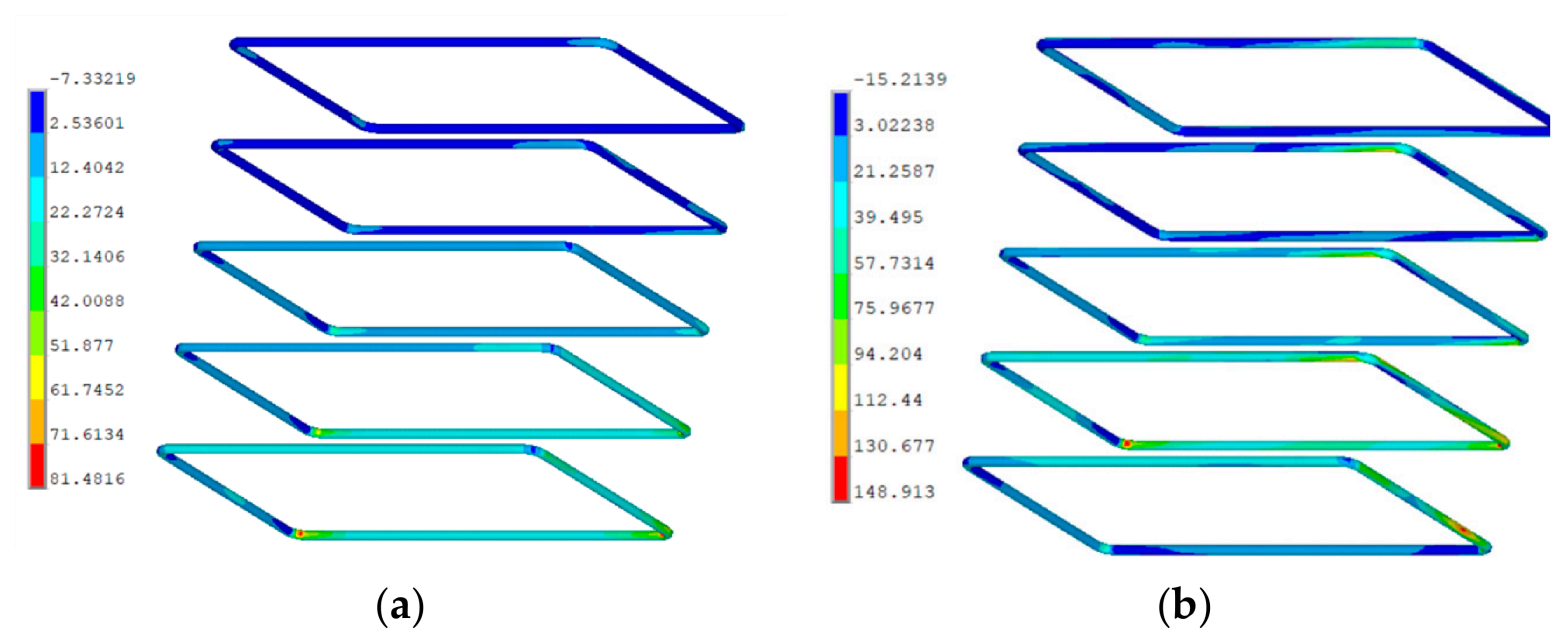
Principal tensile stress distribution of joint FRP stirrups under ultimate load: (**a**) Specimen G31XXX; (**b**) Specimen C31XXX.

Under their respective ultimate loads, the principal tensile stress distributions of the beam longitudinal bars of the two representative specimens (i.e., Specimens G31XXX and C31XXX) are compared in Figure 12, while those for the joint stirrups are compared in Figure 13.

As shown in Figure 12, the principal tensile stress distributions of beam-end longitudinal bars for the two specimens follow the same pattern. The maximum values for Specimens G31XXX and C31XXX occurred at the beam–column interface and were 191.6 MPa and 417.4 MPa, respectively. However, the values remain far below their corresponding tensile strengths. This confirms that the beam-end flexural failure is governed by concrete crushing in the compression zone.

As shown in Figure 13a, the maximum principal tensile stress for the stirrups at the joint panel of Specimen G31XXX is only 81.5 MPa, which is consistent with the relatively intact joint panel under the beam-end flexural failure mode [see Figure 8a]. By contrast, Specimen C31XXX reaches 148.9 MPa [Figure 13b], which reflects the influence of the extensive cracking at the joint panel [see Figure 8b]. Nevertheless, the overall stress level of the joint stirrups of specimen C31XXX still remains relatively low and controllable. It indicates that beam-end bending played a dominant role in the flexural-shear failure mode of this specimen.

**Figure 14:**
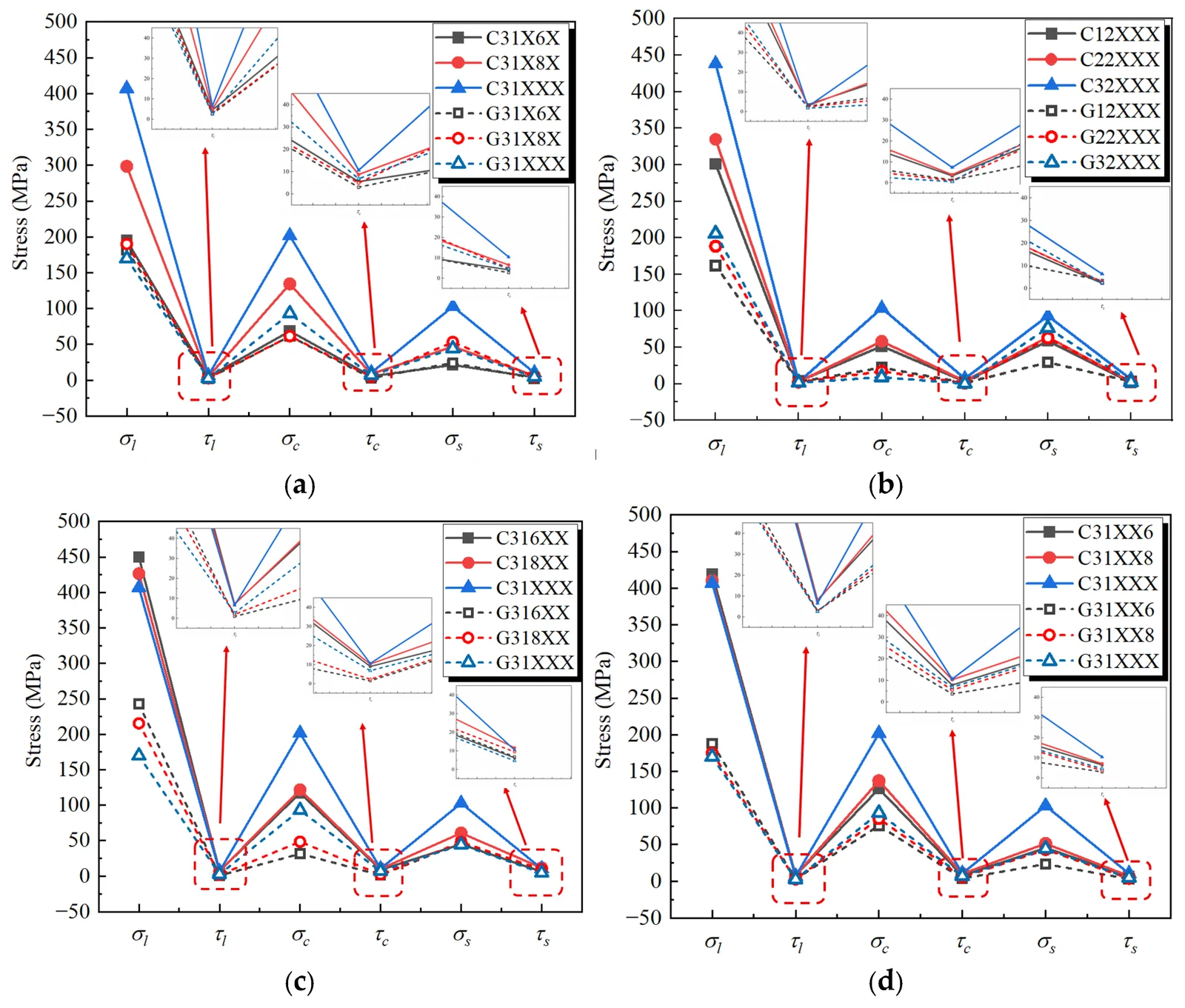
Stress state of FRP reinforcements at critical sections: (**a**) Groups G31X*X and C31X*X; (**b**) Groups G*2XXX and C*2XXX; (**c**) Groups G31*XX and C31*XX; (**d**) Groups G31XX* and C31XX*.

More comprehensively, under the ultimate load, the different stress components of FRP reinforcements at critical sections, expressed in terms of the maximum axial stress and maximum shear stress of beam-end longitudinal bars (*σ*_lb_ and *τ*_lb_), the maximum axial stress and maximum shear stress of joint stirrups (σ_sj_ and τ_sj_) and the maximum axial stress and maximum shear stress of beam-end stirrups (σ_sb_ and τ*_sb_*), were compared among the specimens in different groups and between Series G and C, as shown in Figure 14a–d.

As shown in Figure 14, at the beam end of each of the specimens, the maximum tensile stress of longitudinal bars is significantly higher than that of stirrups. Considering the linear elastic behavior of FRP reinforcements, this reflects the characteristic of flexural failure. With a few exceptions, CFRP reinforcements exhibited significantly higher efficiencies in load response. The maximum tensile stress values of beam longitudinal bars in Groups C31X*X, C*2XXX, C31*XX and C31XX* are 1.1 to 2.1 times, 1.8 to 2.1 times, 1.9 to 2.4 times and 2.2 to 2.4 times those of their GFRP counterparts, respectively. Correspondingly, significantly higher tensile stress values were also recorded for joint CFRP stirrups. This is clearly due to the higher joint horizontal shear force induced by the substantially increased tensile force of beam longitudinal CFRP bars. It should also be noted that the shear stress of the joint stirrups of the CFRP-RC specimens reached a maximum value of 14.6 MPa. As a transversely isotropic material, the epoxy resin matrix has a significant influence on the shear capacity of FRP reinforcements themselves. Considering that the shear strength of epoxy itself is typically in the range of 45 MPa to 50 MPa [67], the shear stress subjected to FRP reinforcements themselves cannot be neglected when reasonably evaluating the axial strength of the FRP reinforcements with complex stress conditions.

### 3.5. Stiffness Degradation

At the *i*-th loading cycle, the development of damage and the degradation in inelastic behavior of the specimens can be evaluated by secant stiffness (*K*_i_). It is defined by Equation (2) [68,69].

*K*_i_ = (|+*F*_i_| + |−*F*_i_|)/(|+*X*_i_| + |−*X*_i_|) (2)

where +*F*_i_ and −*F*_i_ are the peak loads reached in the forward and reverse directions of the *i*-th loading cycle, respectively; +*Δ*_i_ and −*Δ*_i_ are the displacements corresponding to +*F*_i_ and −*F*_i_, respectively.

**Figure 15:**
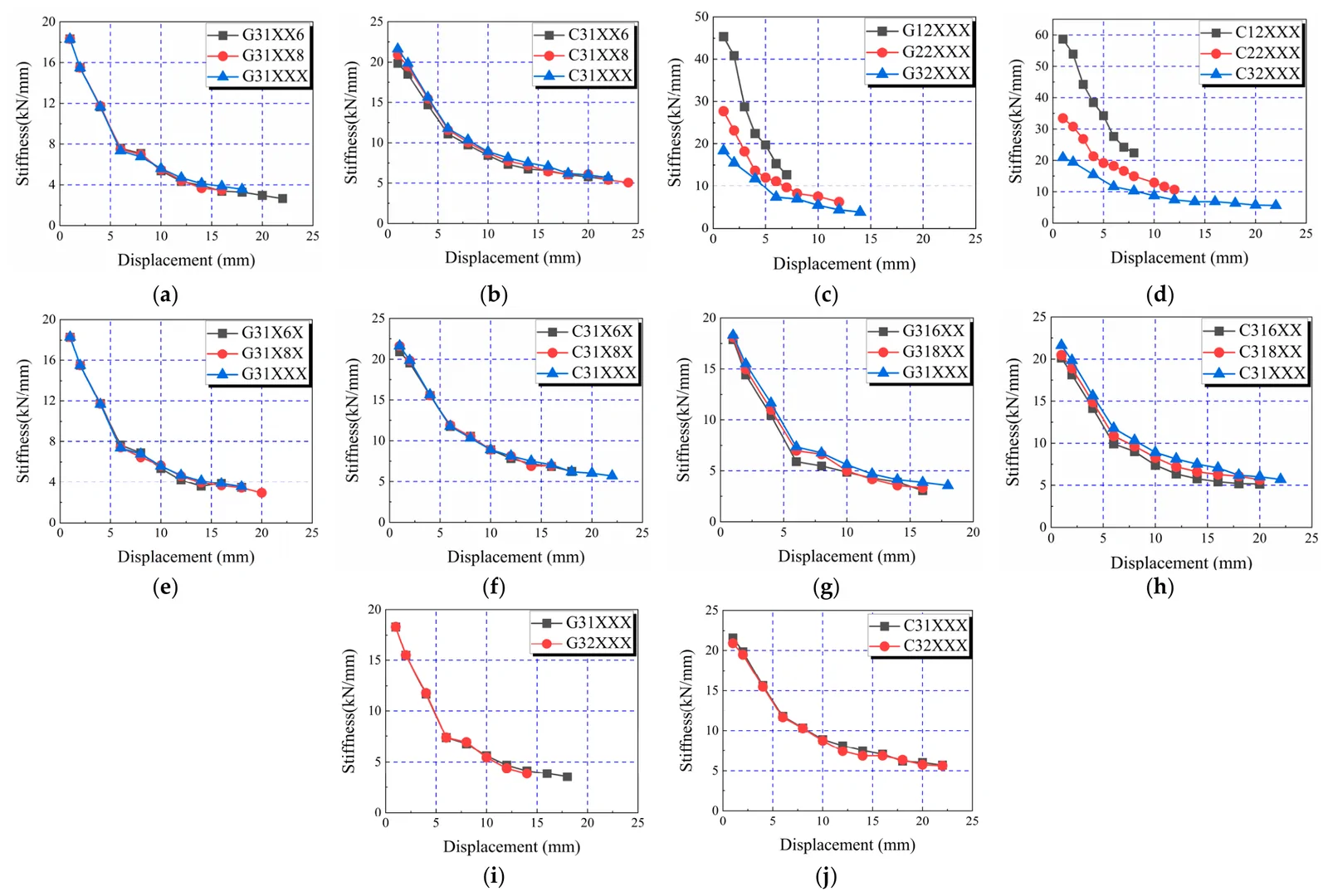
Stiffness degradation: (**a**) Group G31XX*; (**b**) Group C31XX*; (**c**) Group G*2XXX; (**d**) Group C*2XXX; (**e**) Group G31X*X; (**f**) Group C31X*X; (**g**) Group G31*XX; (**h**) Group C31*XX; (**i**) Group G3*XXX; (**j**) Group C3*XXX.

The stiffness degradation curves are compared to each other in the different groups, as shown in Figure 15a–j. Except for Groups G*2XXX and C*2XXX related to the parameter of beam shear–span ratio, the stiffness degradation curves exhibit a two-stage characteristic, as shown in Figure 15a–j. The first stage roughly corresponds to the beam-end displacements of no more than 6 mm. At this stage, the stiffness degradation coefficient decreases rapidly with beam-tip control displacement. It is the consequence of the transition from an elastic state to a nonlinear one. During this process, concrete cracking played a decisive role, as shown in Figure 8. As beam-tip control displacement further increases, the stiffness degradation curves gradually flatten out, which reflects the bending-dominated failure mode at the beam end. On the other hand, as shown in Figure 15c,d, the stiffness degradation curves of Groups G*2XXX and C*2XXX gradually transition from the two-segment form at the beam shear–span ratio of 3.4 to a single-segment one at the beam shear–span ratio of 1.7. Especially, in the single-segment case, the stiffness degradation coefficient decreases rapidly throughout the entire loading process. This is because tat the beam-end failure mode was gradually dominated by shear damage.

Moreover, in the groups associated with the parameters of joint stirrup diameter (Groups G3XX* and C31XX*), joint stirrup spacing (Groups G3*XX and C3*XX) and beam-end stirrup diameter (Groups G31X*X and C31X*X), the stiffness degradation curves overlap each other with no significant differences, as shown in Figure 15a,b,e,f,i,j. This is due to the fact that the failure modes of the specimens in each of the groups were essentially the same.

As shown in Figure 15g,h, in Groups G31*XX and C31*XX, the secant stiffness *K*_i_ increased with the diameter of beam longitudinal bars at each of the beam-tip control displacements. This is due to the enhancement in beam-end bending stiffness from a larger diameter of longitudinal bars (as detailed in Section 3.6).

Especially, CFRP reinforcement was found to be more effective in mitigating the stiffness degradation of FRP-RC beam–column joints. For the first stage of the curves in Groups G31X*X, G31*XX, G31XX* and G3*XXX, the percentage reductions in secant stiffness range from 0.58 to 0.60, from 0.60 to 0.67, from 0.59 to 0.64 and from 0.56 to 0.60, respectively. The percentage reduction was calculated as 1-*K*_IE_/*K*_1_, where *K*_IE_ and *K*_1_ were the secant stiffness values corresponding to the last and first points of the first stage of a stiffness degradation curve, respectively. By contrast, noticeably smaller variations were observed in the CFRP-RC case. The corresponding ranges for Groups C31X*X, C31*XX, C31XX* and C3*XXX are from 0.44 to 0.46, from 0.46 to 0.51, from 0.44 to 0.51 and from 0.40 to 0.46, respectively, i.e., the mean percentage reductions in secant stiffness for Groups C31X*X, C31*XX, C31XX* and C3*XXX are only 0.76, 0.76, 0.77 and 0.74 times their respective GFRP-RC counterparts.

The above results could be attributed to the fact that the stiffness loss due to concrete cracking and crushing was more effectively compensated by the increase in the strain of the FRP reinforcements with a higher elastic modulus (i.e., CFRP reinforcements).

### 3.6. Effects of Parameters

**Figure 16:**
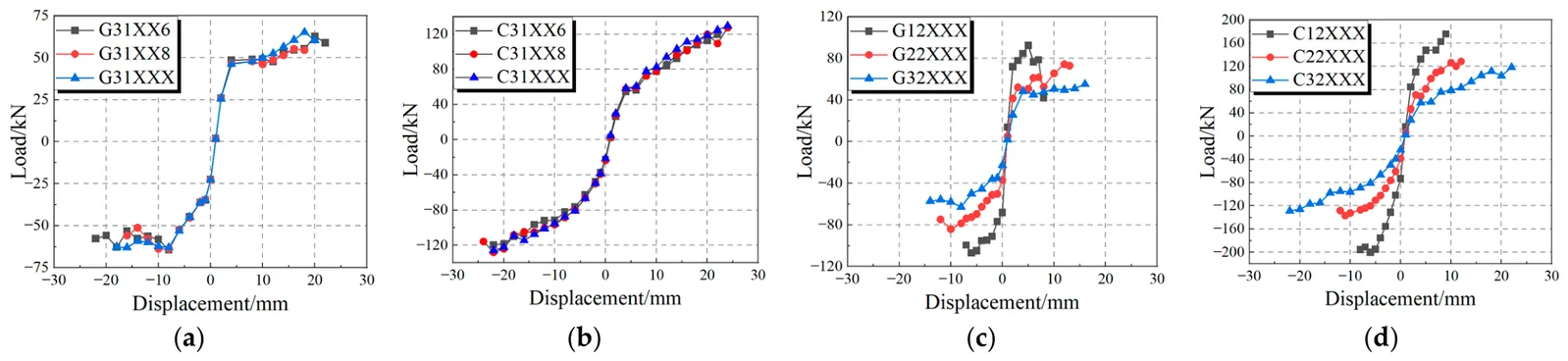

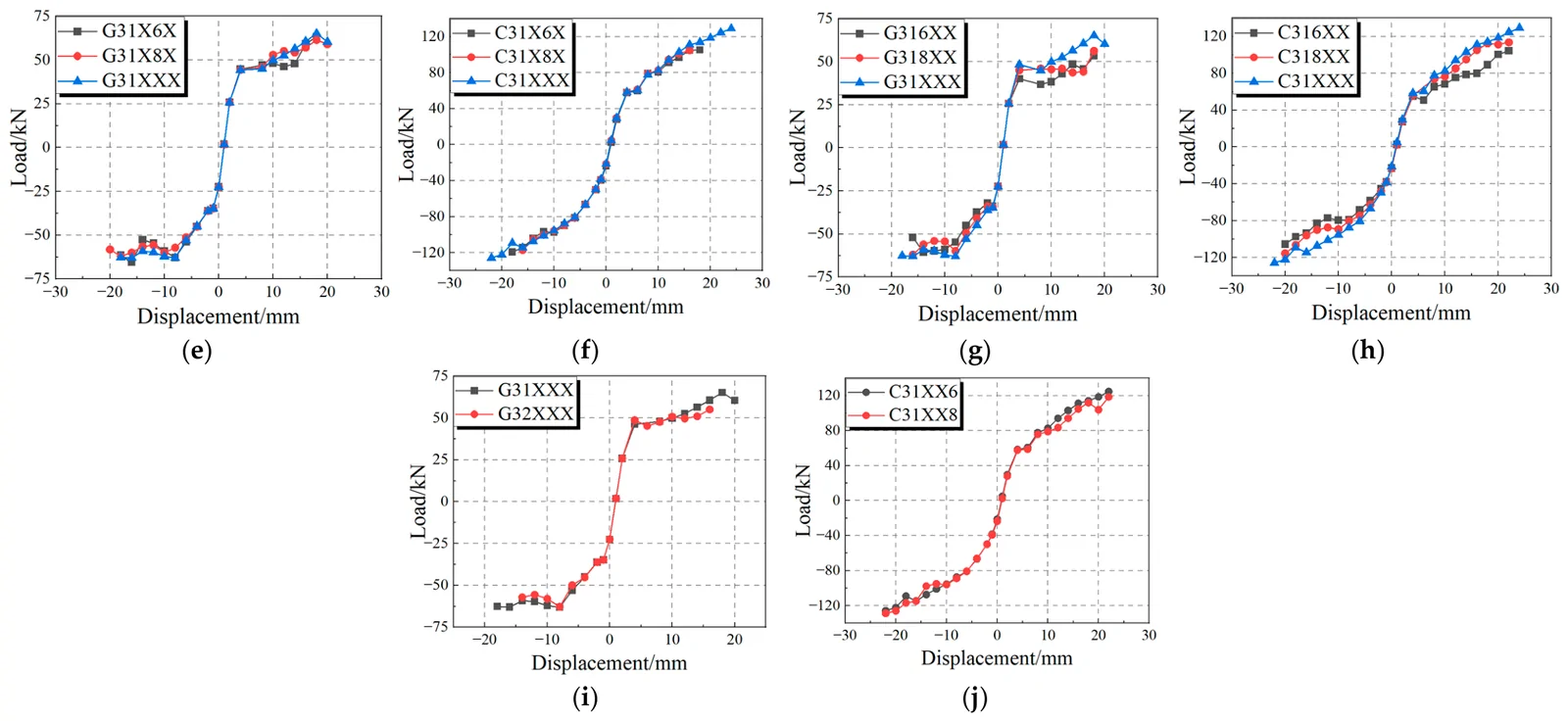
Comparison of the envelope curves in different groups: (**a**) Group G31XX*; (**b**) Group C31XX*; (**c**) Group G*2XXX; (**d**) Group C*2XXX; (**e**) Group G31X*X; (**f**) Group C31X*X; (**g**) Group G31*XX; (**h**) Group C31*XX; (**i**) Group G3*XXX; (**j**) Group C3*XXX.

Besides the stiffness degradation case (detailed in Section 3.5), the differences among the effects of different parameters are clearly reflected by the envelope curves of the specimens. The envelope curves were compared in the different groups, as shown in Figure 16a–j.

As shown in Figure 16, the significant differences among the roles of the parameters were revealed. Furthermore, the parameters can be classified into three categories according to the sensitivity of load response to them. The assessment of the sensitivity for a specific parameter was performed in the specimen groups where only the value of the parameter changed. The first category refers to relatively low sensitivity and involves only two parameters, i.e., joint stirrup diameter (Groups G31XX* and C31XX*) and joint stirrup spacing (Groups G*2XXX and C*2XXX). The second category includes parameters with obvious effects, namely beam-end stirrup diameter (Groups G31X*X and C31X*X) and beam longitudinal bar diameter (Groups G31*XX and C31*XX). The third category consists of those with significant impacts, namely FRP type (Series C and G) and beam shear–span ratio (Groups G3*XXX and C3*XXX).

As shown in Figure 16a,b,i,j, the diameter and spacing of the stirrups at the joint panel had the least influence on the envelope curves. The envelope curves in the four groups overlap each other, while limited differences exist in the vicinities of their respective ultimate loads. This confirms that the failure of the related specimens was dominated by that of their beam ends. That is, a reasonable strength hierarchy was achieved for the different components of both CFRP-RC and GFRP-RC beam–column joints. As a result, further increasing the ratio of joint stirrups provided a limited improvement in ultimate load and stiffness degradation. It should also be noted that the larger the ratio of joint stirrups was, the stronger the concrete confinement at the joint panel was, and the greater the beam-end rotational stiffness at the beam–column interface was. This is the reason for the minor differences among the envelope curves.

On the other hand, the ultimate loads of the specimens in Groups G31X*X and C31X*X increased with the diameter of the beam-end stirrups, as shown in Figure 16e,f. This implies that the stronger confinement provided by stirrups was beneficial for confining the beam-end compression zone. As a result, the propagation of concrete cracks was suppressed, and the crushing of the compression zone was delayed. In such cases, the improvements in load responses of beam–column joints came from the enhanced flexural capacity of the confined beam ends. Furthermore, the effects of the parameter on Groups G31X*X and C31X*X differed to some extent. For the GFRP-RC specimens, as the beam-end stirrup diameter was increased from 6 mm to 10 mm, the variations among the envelope curves were relatively small. Only were limited improvements in ultimate load recorded. By contrast, their CFRP-RC counterparts exhibited better improvements not only in ultimate load but also in deformation capacity. Especially, as the beam-end stirrup diameter was increased from 8 mm to 10 mm, the ultimate displacement significantly increased by 50%.

As shown in Figure 16g,h, the diameter of the beam longitudinal bars had a more noticeable influence on the envelope curves in Groups G31*XX and C31*XX. It was noted that increasing the diameter of beam longitudinal bars significantly enhanced the ultimate load. The increases in ultimate loads in Groups G31*XX and C31*XX were up to 21.8% and 13.3%, respectively, as the diameter was increased from 16 mm to 20 mm. This is a natural outcome of the bending-dominated failure mode of the beam end. In particular, the increased bending stiffness of the beam end from a larger diameter of longitudinal bars was beneficial for achieving a higher bending moment before the compression zone was crushed under excessive rotation. Moreover, in Group G31*XX, the increase in ultimate load primarily occurred as the diameter was increased from 18 mm to 20 mm. On the other hand, in Group C31*XX, each change in the diameter from 16 mm to 20 mm resulted in a noticeable increase in ultimate load. Obviously, this is because the bending stiffness of the beam end is more sensitive to changes in the diameter of high-modulus FRP bars.

As shown in Figure 16i,j, the beam-end shear–span ratio had a significant influence on the envelope curves in Groups G3*XXX and C3*XXX. As the shear–span ratio increased from 1.7 to 3.4, both the ultimate load and the initial slope of the envelope curve decreased noticeably. That is, at smaller shear–span ratios, the beam shear force could be transmitted more directly to the joint panel by a diagonal compression mechanism. In turn, it facilitated the formation of a stronger diagonal compression strut mechanism at the joint panel. Hence, the overall stiffness as well as ultimate load of beam–column joints was enhanced. As the shear–span ratio was increased, beam-end deformation became dominated by flexural effects, which led to a decrease in the lateral stiffness of beam–column joints.

In summary, within the scope of this study, FRP type and beam shear–span ratio are the key factors affecting the seismic performance of FRP-RC beam–column joints, due to their directly effects on mechanical behaviors; with the expected bending-dominated failure mode at the beam end, the lateral resistance of beam–column joints is improved to a certain extent by increasing the ratio of longitudinal bars or stirrups at the beam end; once the shear failure at the joint panel is avoided through a properly designed strength hierarchy, the effect of further increasing the ratio of joint stirrups is primarily reflected in controlling the damages at the beam–column interface.

## 4. Development of Hysteretic Model

### 4.1. Fitting of Envelope Curves

**Figure 17:**
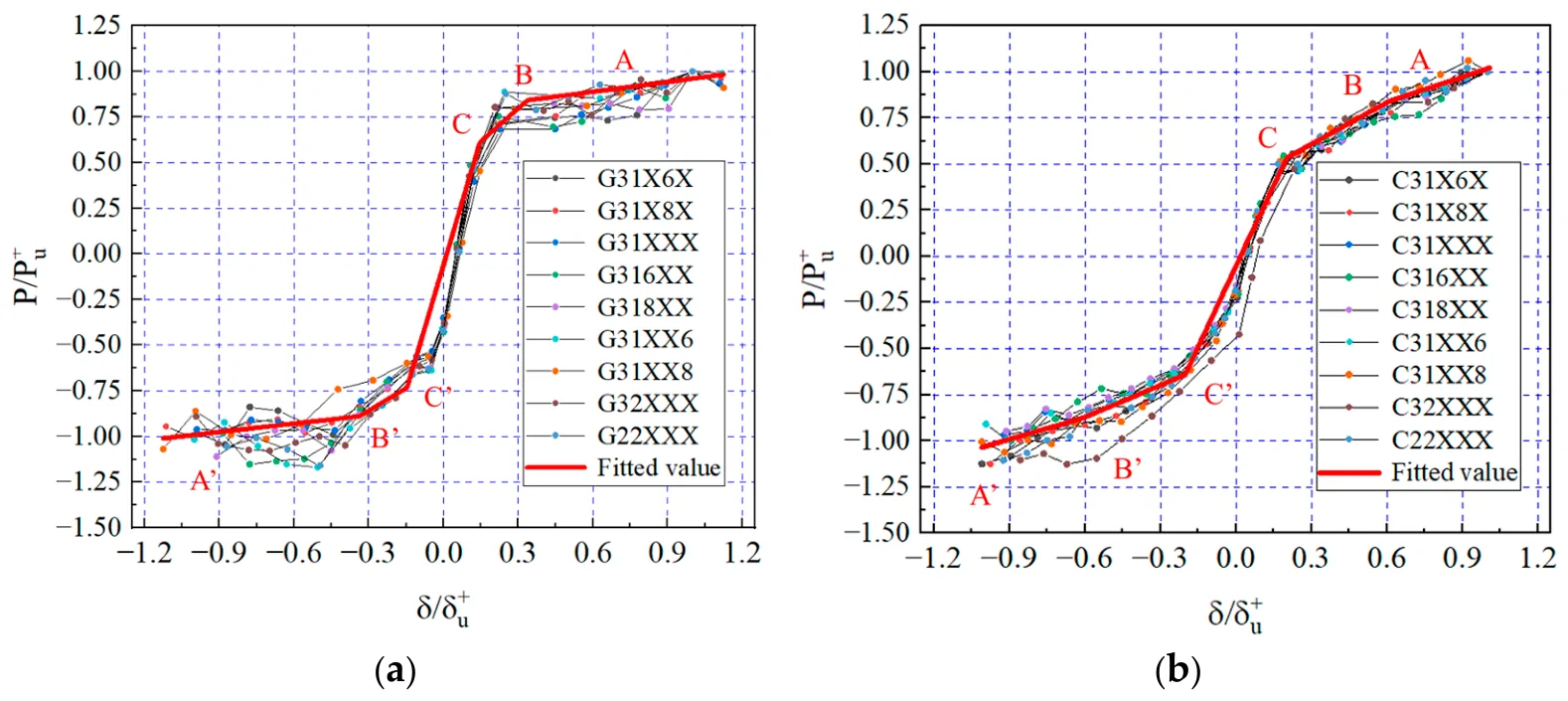
Fitted envelope curve: (**a**) Series G; (**b**) Series C.

The abscissa and ordinate for the points at each of the envelope curves were normalized with beam-tip ultimate load and its corresponding displacement, respectively. The normalized envelope curves for Series G and C are presented in Figure 17a,b, respectively. A fitted envelope curve was obtained from the normalized envelope curves for each of the series.

As shown in Figure 17, the forward and reverse branches of the fitted envelope curves consist of several line segments and are essentially antisymmetric. Furthermore, the fitted envelope curves can be divided into three stages, namely the elastic stage (CC′), nonlinear stage I (BC and B′C′) and nonlinear stage II (AB and A′B′). In the elastic stage CC′, the lateral loads applied on FRP-RC beam–column joints are relatively small. The joint panel and the beam and column ends remain in an elastic state, while the aggregates and cement mortar in concrete deform compatibly without significant cracking. As the lateral load is increased, FRP-RC beam–column joints enter the nonlinear stage I. At this stage, material limits are beginning to be reached by concrete and its interface with FRP reinforcements. Macroscopic cracks occur and propagate at the beam end and joint panel, which leads to a reduction in lateral stiffness. With stress redistribution, the roles of FRP reinforcements are becoming increasingly prominent, especially at the tensile zone of the beam end. As a result, the load continues to increase relatively slowly. Finally, at the nonlinear stage II, stiffness degradation is even more severe. Owing to the linear elastic behavior of FRP reinforcements, the load continues to increase. However, the secant stiffness of the fitted envelope curve drops rapidly.

As shown in the comparison between Figure 17a,b, at the first stage, the average initial stiffness values of the fitted envelope curves for GFRP-RC and CFRP-RC beam–column joints are relatively close. This confirms that the load response during this stage is primarily controlled by concrete. At the latter two stages, the slopes of the fitted envelope curve for CFRP-RC beam–column joints are markedly higher than their GFRP-RC counterparts. Evidently, not only FRP reinforcements but also the types of their fibers play an important role. First, the beam longitudinal CFRP bars, with a much higher elastic modulus in the fiber direction, provide a greater contribution to load response. Second, the beam end is confined more effectively by CFRP stirrups. Hence, the concrete degradation at the compression zone is delayed. Therefore, the FRP type should be considered in developing the hysteretic model for FRP-RC beam–column joints.

Due to the asymmetry in the fitted envelope curves, only the mathematical model for the forward/positive branch was developed. According to their shape characteristics, the fitted envelope curve of FRP-RC beam–column joints can be represented by a trilinear model, as expressed in Equation (3).

(3) $$P(x)=\{\begin{array}{ll} k_{e}x, & 0\leq x\leq x_{D}\\ P_{D}+k_{d1}(x-x_{D}), & x_{D}<x<x_{E}\\ P_{E}+k_{d2}(x-x_{E}), & x_{E}<x<1 \end{array}$$

where *P* and *x* are defined as *P*/*P*_max_ and *δ*/*δ*_max_, respectively; *P*_max_ and *δ*_max_ are beam-tip ultimate load and the corresponding displacement; *k*_e_, *k*_d1_ and *k*_d2_ denote the slopes of the elastic stage, nonlinear stage I and nonlinear stage II, respectively; for GFRP-RC beam–column joints, *k*_e_, *k*_d1_ and *k*_d2_ are 4.6, 0.84 and 0.15, respectively; for CFRP-RC beam–column joints, *k*_e_, *k*_d1_ and *k*_d2_ are 2.93, 0.59 and 0.45, respectively; *P*_D_ and *P*_E_ denote the maximum normalized loads reached at the ends of the elastic stage and nonlinear stage I, respectively; and *x*_D_ and *x*_E_ are the normalized displacements related to *P*_D_ and *P*_E_, respectively.

### 4.2. Stiffness Degradation Model

**Figure 18:**
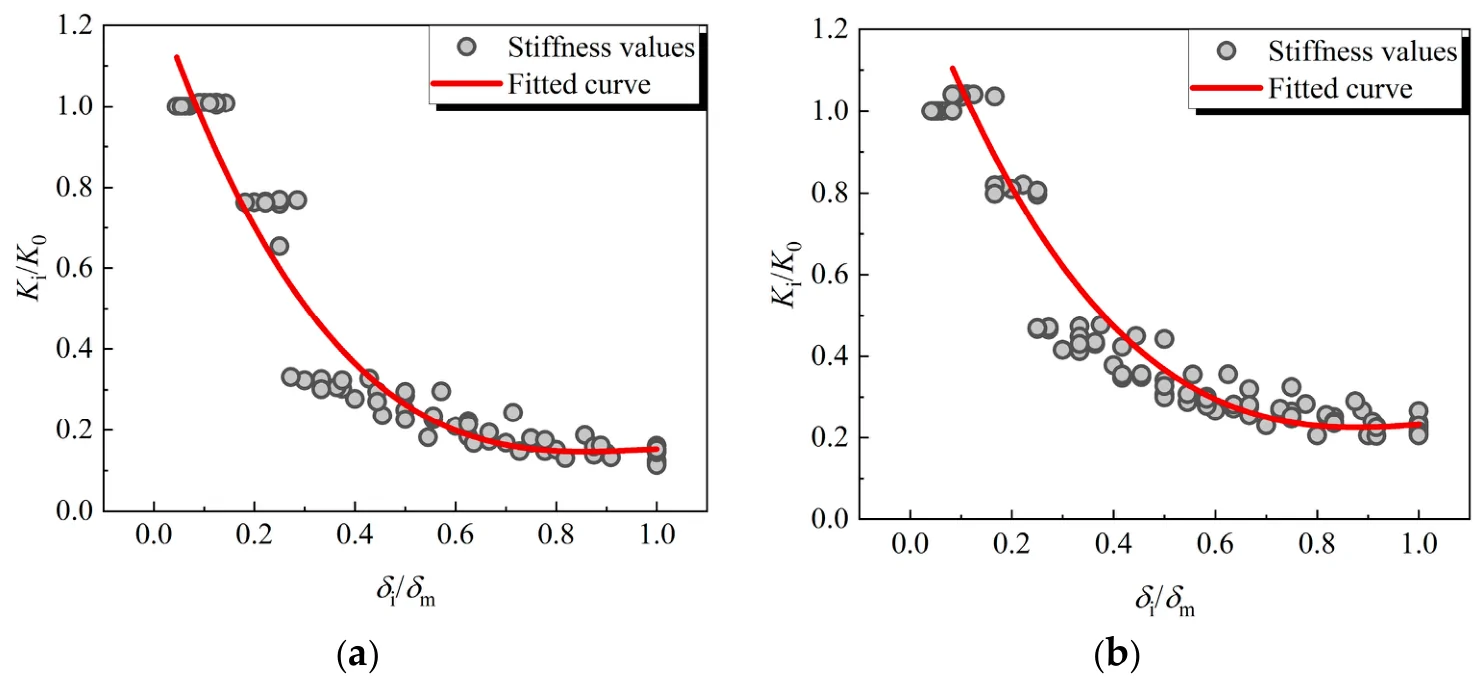
Regression curves for normalized secant stiffness: (**a**) Series G; (**b**) Series C.

While the loading branch of the hysteresis model is obtained geometrically from the fitted envelope curve (as detailed in Section 4.3), it is equally important to determine the slope of the unloading branch to accurately reconstruct the shape of hysteretic loops. Especially, based on the classical Pivot rule [70,71], a regression method was employed to obtain the dimensionless analytical relationship between the unloading slope and the beam-tip displacement. Considering the significant differences between the seismic performance of the CFRP-RC and GFRP-RC beam–column joints, nonlinear regression analyses were performed separately for Series G and C, as shown in Figure 18a,b. The numerical results of the specimens with the beam shear–span ratio of 3.4 were selected. At this ratio, the expected bending-dominated failure mode is more likely to occur. The regression formulas obtained for Series G and C are presented in Equation (4), respectively.

(4) $$y=\{\begin{array}{ll} 1.3-3.5x+3.6x^{2}-1.2x^{3}, & \mathrm{GFRP}-\mathrm{RC}\\ 1.4-3.3x+3.2x^{2}-x^{3}, & \mathrm{CFRP}-\mathrm{RC} \end{array}$$

where *x* and *y* are defined as *K*_i_/*K*_0_ and *δ*_i_/*δ*_max_, respectively; *K*_0_ is the initial stiffness corresponding to the elastic stage; *K*_i_ and *δ*_i_ are the secant stiffness and the beam-tip displacement of the *i*–th load step; *δ*_max_ is the beam-tip displacement related to the ultimate load.

As shown in Figure 18 and Equation (4), the cubic polynomial can accurately reflect the stiffness degradation behavior of FRP-RC beam–column joints under cyclic loading. It is noted that significant dispersion is exhibited by the data points, which explains why a linear or quadratic polynomial is not suitable for this regression. On the other hand, although a similar trend is presented by the regression curves for two types of FRP-RC beam–column joints, the regression curve for GFRP-RC beam–column joints decreases more sharply with beam-tip displacement.

### 4.3. Hysteretic Model

**Figure 19:**
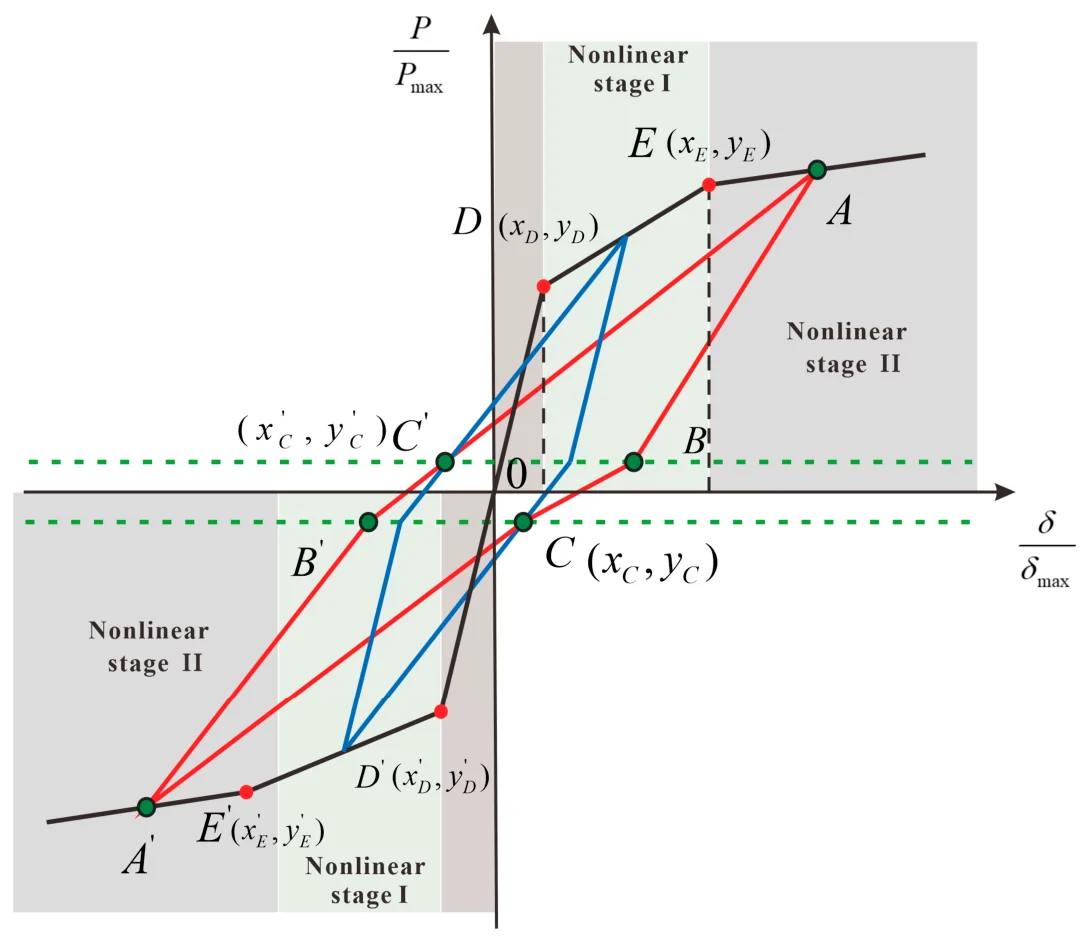
Hysteretic model.

When FRP-RC beam–column joints are in the elastic stage, both the loading and unloading branches of the hysteretic model extend along Segment OD, as illustrated in Figure 19. Once the specimen enters the nonlinear state (i.e., the nonlinear stage I), the pronounced hysteretic behavior is characterized by a significant difference between the slopes of the loading and unloading branches. The geometric-path method is adopted to establish a complete hysteretic model for FRP-RC beam–column joints [72]. Based on the fitting of the FE hysteretic curves, the control points of C and C′ are obtained. These points provide the additional geometric constraints for the loading branches in the forward/positive and reverse/negative directions. Hence, the numerical stability of the hysteretic model is enhanced. Based on the fitted envelope curves (detailed in Section 4.1), the stiffness degradation model (detailed in Section 4.2) and control points (including but not limited to Points C and C′), the loading and unloading paths are determined by the method described below.

First, the fitted envelope curve [i.e., Equation (3)] is used to establish the loading paths under different beam-tip control displacements. The control points A and A′ are those on the fitted envelope curve and correspond to the forward and reverse control displacements of a given hysteresis cycle, as shown in Figure 19. During forward loading, the loading path extends from Point C′ to Point A, whereas the reverse loading path is from Point C to Point A′.

Second, once the loading branch reaches the fitted envelope curve, unloading is triggered along the path from Point A to Point B. The slope of the unloading branch (i.e., Segment AB) is directly related to the extent of stiffness degradation. That is, the slope is determined according to the stiffness degradation model [Equation (4)]. According to the geometric-path method [72], the control point B is the intersection of Line *y* = *y*_C_′ and the unloading branch. The reverse loading is achieved with a transitional path from Point B to Point C. The subsequent loading and unloading paths in the reverse direction are similar to their respective positive counterparts.

**Figure 20:**
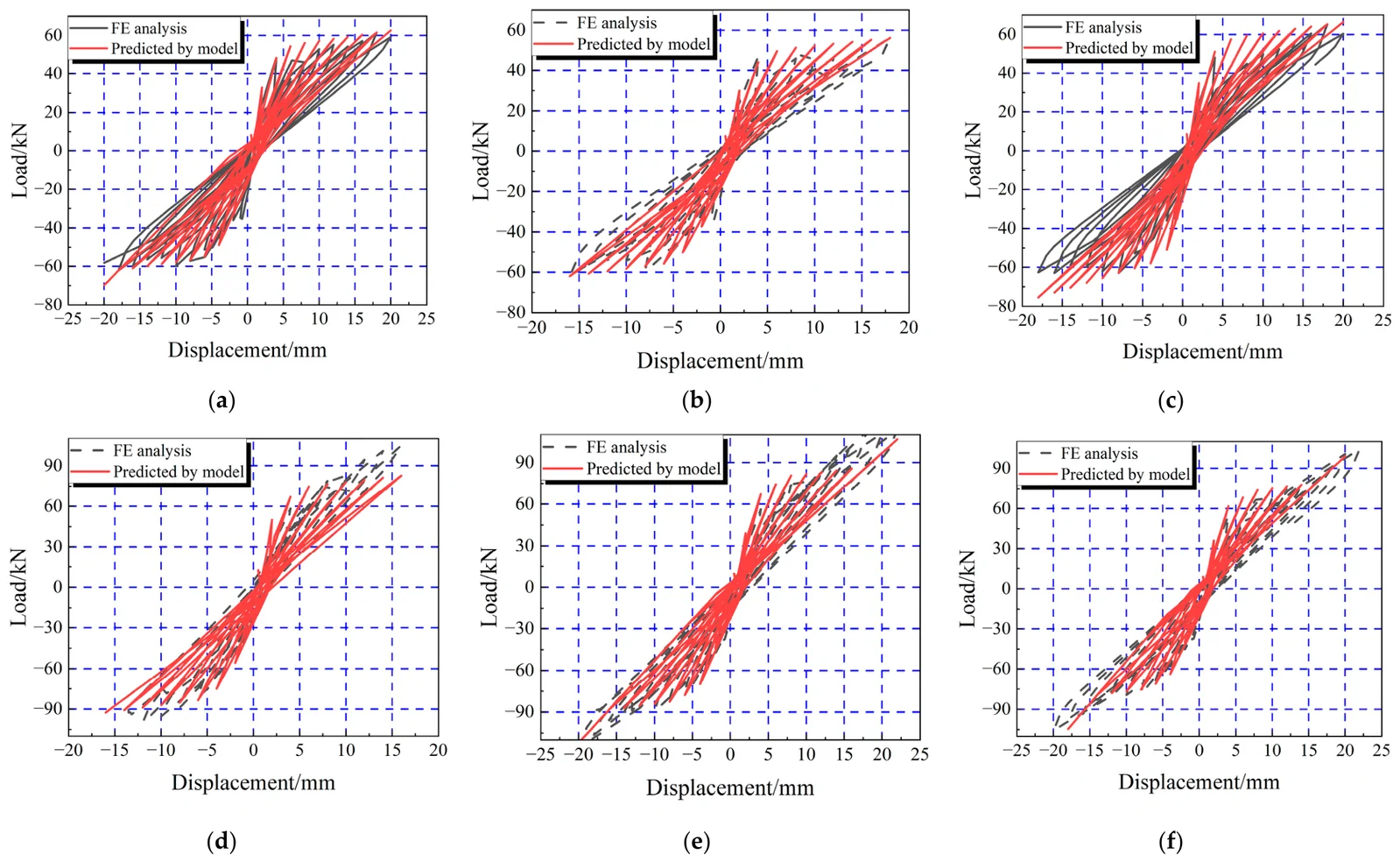
Comparison between hysteretic curves predicted by proposed hysteretic model and their FE counterparts: (**a**) Specimen G31X8X; (**b**) Specimen G318XX; (**c**) Specimen G31XXX; (**d**) Specimen C31X8X; (**e**) Specimen C318XX; (**f**) Specimen C31XXX.

In each of Series G and C, the coordinates of the control points are obtained from the fitting analysis of the hysteretic curves from FE analysis, as listed in Table 4. To verify the accuracy of the proposed hysteretic model, the predicted hysteretic curves were compared with their FE counterparts, as shown in Figure 20a–f. A total of six specimens were selected from Series G and C. As shown in the plots, the hysteretic curves predicted by the proposed hysteretic model agree well with those obtained from FE analysis. Therefore, the proposed hysteretic model can be used for the nonlinear seismic analysis of FRP-RC beam–column joints as well as frame structures.

## 5. Conclusions

In this study, a refined three-dimensional nonlinear finite element modeling approach was developed to clarify the roles of a series of parameters on the seismic performance of FRP-RC beam–column joints. The main conclusions could be summarized in a rather simplified manner as follows:

(1) The failure of the rationally designed FRP-RC beam–column joints was governed by beam-end flexural damage, achieving the strength hierarchy required for seismic-resistant frames. It confirms the feasibility of replacing conventional steel reinforcements with transversely isotropic and linear elastic FRP ones.
(2) The type of FRP reinforcements has the most significant impact on the seismic performance of FRP-RC beam–column joints. Compared with their GFRP-RC counterparts, the ultimate loads of CFRP-RC specimens increased by up to 92.3%, accompanied by a 22.2% increase in the corresponding beam-end displacement.
(3) The unexpected excessive joint shear force was caused by the beam longitudinal CFRP bars due to their relatively high elastic modulus. Besides force equilibrium, deformation compatibility should be considered in establishing the strength hierarchy for the different components of an FRP-RC beam–column joint.
(4) As disclosed by the solid modeling approach, the shear stress suffered by the stirrups themselves reached up to 12.4 MPa and 14.6 MPa at the beam end and the joint panel, respectively. Therefore, given the nature of transverse isotropy, FRP reinforcements should be designed with a tensile-shear strength criterion.
(5) The shear–span ratio of the beam also exhibited a significant influence on the overall performance of the specimens. As the ratio was increased from 1.7 to 3.4, both ultimate load and initial stiffness decreased significantly; meanwhile, the rate of stiffness degradation gradually slowed down.
(6) Increasing the diameter of beam-end longitudinal FRP bars had a direct effect on enhancing the ultimate load of FRP-RC beam–column joints. However, the relationship was far from linear, due to the pseudo-ductile beam-end failure mode of the concrete crushing in the compression zone.
(7) In addition to the improvement in shear, another function of the beam-end FRP stirrups was to provide confinement to the compression zone and delay the crushing of concrete. Hence, even under a flexural failure mode, increasing the diameter of the beam-end stirrups had a positive effect.
(8) Under the beam-end flexural failure, the effect of further increasing the ratio of joint stirrups was limited. However, the larger the ratio of joint stirrups was, the stronger the concrete confinement at the joint panel was, and the greater the rotational stiffness of the beam end at the beam–column interface.
(9) GFRP reinforcements are a suitable choice in the scenarios associated with low-to-moderate seismic demands and stringent cost management. For the structures situated in highly seismic regions or the seismic applications requiring enhanced performances, CFRP ones should be given priority.
(10) Taking the FRP type into account, a hysteretic model that can accommodate degradations in stiffness and strength was developed and verified against FE results. The proposed model provides a basis for the seismic design of FRP-RC beam–column joints as well as frame structures.

## Figures and Tables

**Table 1:** Specimen details and nonlinear FE analysis results.

Series	Specimen ID	Key Parameters					FE Results			
		*d_vj_* (mm)	*S_svj_* (mm)	*d_b_* (mm)	*d_vb_* (mm)	*λ*	*P*_u_^+^ * (kN)	*δ*_u_^+^ * (mm)	*P_u_*^−^ * (kN)	*δ*_u_^−^ * (mm)
**C**	C31X6X	10	100	6	20	3.4	104.4	18.1	−118.8	−17.8
	C31X8X	10	100	8	20	3.4	104.9	16.0	−115.8	−15.9
	C31XXX	10	100	10	20	3.4	128.2	23.8	−125.5	−21.9
	C12XXX	10	350	10	20	1.7	173.6	9.1	−212.4	−7.8
	C22XXX	10	350	10	20	2.6	121.8	12.0	−156.8	−10.9
	C32XXX	10	350	10	20	3.4	115.8	21.9	−128.7	−21.9
	C316XX	10	100	10	16	3.4	103.5	21.9	−104.7	−19.9
	C318XX	10	100	10	18	3.4	113.8	22.1	−113.8	−19.9
	C31XX6	6	100	10	20	3.4	121.5	19.9	−112.0	−17.9
	C31XX8	8	100	10	20	3.4	126.9	24.2	−126.9	−21.9
**G**	G31X6X	10	100	6	20	3.4	62.9	17.8	−65.0	16.0
	G31X8X	10	100	8	20	3.4	61.5	18.0	−61.5	−17.8
	G31XXX	10	100	10	20	3.4	64.7	17.8	−63.2	−15.9
	G12XXX	10	350	10	20	1.7	91.8	5.0	−106.3	−6.0
	G22XXX	10	350	10	20	2.6	73.7	11.9	−82.6	−9.9
	G32XXX	10	350	10	20	3.4	54.8	15.9	−62.5	−8.0
	G316XX	10	100	10	16	3.4	53.9	15.6	−60.0	−14.4
	G318XX	10	100	10	18	3.4	56.3	15.6	−60.9	−16.2
	G31XX6	6	100	10	20	3.4	59.8	14.6	−64.2	−16.5
	G31XX8	8	100	10	20	3.4	55.4	16.1	−63.3	−10.0

* *P*_u_^+^, *P_u_*^−^, *δ*_u_^+^, and *δ*_u_^−^ are the ultimate loads and the corresponding displacements reached in forward and reverse directions.

**Table 2:** Engineering constants for FRP reinforcements.

Type	Elastic Modulus (MPa)			Poisson’s Ratio			Shear Modulus (MPa)		
	*E*_x_	*E*_Y_	*E*_Z_	*ρ*_XY_	*ρ*_YZ_	*ρ*_XZ_	*GXY*	*GXZ*	*GYZ*
CFRP	140,600	8549	8549	0.019	0.41	0.019	4799	4799	3033
GFRP	40,000	8000	8000	0.015	0.35	0.015	4000	4000	1567

**Table 3:** Comparison between FE and experimental results.

	Specimen JFRP1			Specimen GG-3		
	Test	FE	FE/Test	Test	FE	FE/Test
Average maximum load (kN)	124.1	127.1	1.02	180.9	173	0.96
Average displacement at maximum load (mm)	77	76	0.99	93.7	108.8	1.16

**Table 4:** Coordinates of control points in hysteretic model.

	*x*_D_	*x*_E_	*x*_C_	*y*_C_	*x*_C_′	*y*_C_′
GFRP-RC joints	0.15	0.35	1.52	–3.93	0.90	3.47
CFRP-RC joints	0.22	0.62	1.14	–6.87	0.77	5.12

## Data Availability

The original contributions presented in this study are included in the article. Further inquiries can be directed to the corresponding author.
